# Theranostic application of radiolabeled HER2-targeting single-domain antibodies (RAD202) in preclinical setting

**DOI:** 10.1038/s41598-025-25679-w

**Published:** 2025-11-04

**Authors:** Betül Altunay, Luka Obradović, Alexandru Florea, Andreas T. J. Vogg, Laura Schäfer, Agnieszka Morgenroth, Levente K. Meszaros, Fabian Hertel, Malik E. Juweid, Felix M. Mottaghy

**Affiliations:** 1 Department of Nuclear Medicine, Uniklinik RWTH Aachen, 52074 Aachen, Germany; 2 Radiopharm Theranostics Ltd, 62 Lygon Street, Carlton South, VIC 3053 Australia; 3 Center of Integrated Oncology (CIO), Universities of Aachen, Bonn, Cologne and Düsseldorf, Kerpener Str. 62, 50937 Cologne, Germany; 4 Department of Radiology and Nuclear Medicine, University of Jordan, Amman, Jordan; 5 Department of Radiology and Nuclear Medicine, Maastricht University Medical Centre, Maastricht, The Netherlands

**Keywords:** HER2, Nanobody, Single domain antibody, HIS-tag, RAD202, Cancer, Drug discovery, Oncology

## Abstract

HER2 is a well-established target for antibody-based drug delivery. Single-domain antibodies (sdAbs) offer advantages over monoclonal antibodies, including faster clearance, higher antigen-binding affinity, and improved tumor penetration. This study investigates the biodistribution of HER2-targeting sdAbs (with and without HIS-tag) labeled with ^68^Ga and their therapeutic efficacy radiolabeled with ^177^Lu, in HER2-positive xenograft. Mice received 10 MBq of ^68^Ga-labeled HIS-tagged sdAb and underwent PET/CT scans immediately and three hours post-injection. The following day, untagged radioactive sdAb was administered and scanned similarly. Controls were co-injected with an excess of unlabeled sdAb. For therapy study, mice received either four weekly injections or a single dose of ^177^Lu-labeled sdAb (without HIS-tag). Radiotracers were synthesized with high radiochemical purity (> 99.8%). PET/CT imaging revealed specific tumor accumulation and low background, except in excretory organs. Co-injection of unlabeled sdAbs reduced tumor uptake, confirming tracer specificity. ^177^Lu-labeled sdAb therapy prolonged median survival by 34 d (single dose) and 26 d (fractionated therapy) versus respective control group treated with unlabeled sdAb. The fractionated therapy group exhibited significantly lower tumor volumes and did not exceed 200% of the initial tumor volume. ^68^Ga-labeled sdAbs enabled high-contrast PET imaging and ^177^Lu-labeled sdAbs, particularly in fractionated regimens, effectively delayed tumor progression and were well tolerated, supporting further clinical translation.

**Supplementary Information:**

The online version contains supplementary material available at 10.1038/s41598-025-25679-w.

## Introduction

Alterations in human epidermal growth factor receptor 2 (HER2) expression have been observed in various cancer types, such as those arising in the breast, gastrointestinal tract, urinary bladder, salivary glands, lung, ovaries, colon, and pancreas. Changes in HER2 expression in tumor cells are mainly caused by mutations, amplifications or overexpression of the HER2 gene (erbB2). HER2 overexpression is associated with aggressive behavior, poor prognosis and shorter overall survival^1,2^.

In this context, the development of HER2-targeted radiotracers is essential for non-invasive assessment of HER2 expression, enabling patient selection for HER2-targeted therapies, monitoring treatment response, and detection of arising resistance. While various probes, particularly antibodies, have been developed for HER2 imaging, radiolabeled single-domain antibodies (sdAbs) have recently gained attention for their potential in this field. These sdAbs are the smallest antigen-binding domains of heavy chain-only camelid antibodies. They are ideal for radioimmunoimaging due to their small size, low immunogenicity, rapid blood clearance, and high antigen affinity^2^. However, the main drawback of radiolabeled sdAbs for in vivo application is their high kidney accumulation due to renal elimination. Their small size allows filtration and reabsorption in the renal cortex, increasing the risk of nephrotoxicity from radiation^3^. Studies have shown that positively charged amino acids or Gelofusine^®^ can reduce renal retention of peptides and small proteins. Gelofusine^®^, a succinylated bovine gelatin used as a plasma expander, increases the excretion of megalin ligands, competitively displacing radiolabeled proteins and lowers kidney retention^4^.

In a previous study, we radiolabeled a HER2-targeting sdAb, NM-02^5^, *via* its C-terminal hexahistidine tag (HIS-tag) with technetium-99m (^99m^Tc-RAD201). The radiotracer has shown safety, favorable biodistribution, and effective imaging in HER2-positive tumor sites, including visceral, lymph node, skeletal, and intracranial lesions. Using a plasma expander reduced kidney radiation dose by nearly 50% without compromising tumor uptake. ^99m^Tc-RAD201 exhibited strong tumor targeting and rapid blood clearance, enabling SPECT/CT imaging just hours after injection, even during “cold” HER2-targeted therapy^5,6^.

Here, we radiolabeled the HER2-targeting sdAbs, NM-02 (with C-terminal HIS-tag) and its second-generation variant NM-02.1 (without C-terminal HIS-tag), with Gallium-68 and Lutetium-177 *via* DOTAGA as a chelator and investigated regarding biodistribution and therapeutic effect in HER2-positive xenografts.

## Results

### Synthesis of and in vitro studies with [^68^Ga]Ga-DOTA-GA-RAD202 and [^68^Ga]Ga-DOTA-GA-RAD202.1

Radiosynthesis of and in vitro studies with [^68^Ga]Ga-DOTA-GA-RAD202 and [^68^Ga]Ga-DOTA-GA-RAD202.1 can be found in the Supplemental Data (Figures S1-S3). The [^68^Ga]Ga-DOTA-GA-sdAb tracers were synthesized with high radiochemical purity (RCP, 99.8 ± 0.5%).

### PET imaging and biodistribution studies with [^68^Ga]Ga-DOTA-GA-RAD202 and [^68^Ga]Ga-DOTA-GA-RAD202.1

**Fig. 1:**
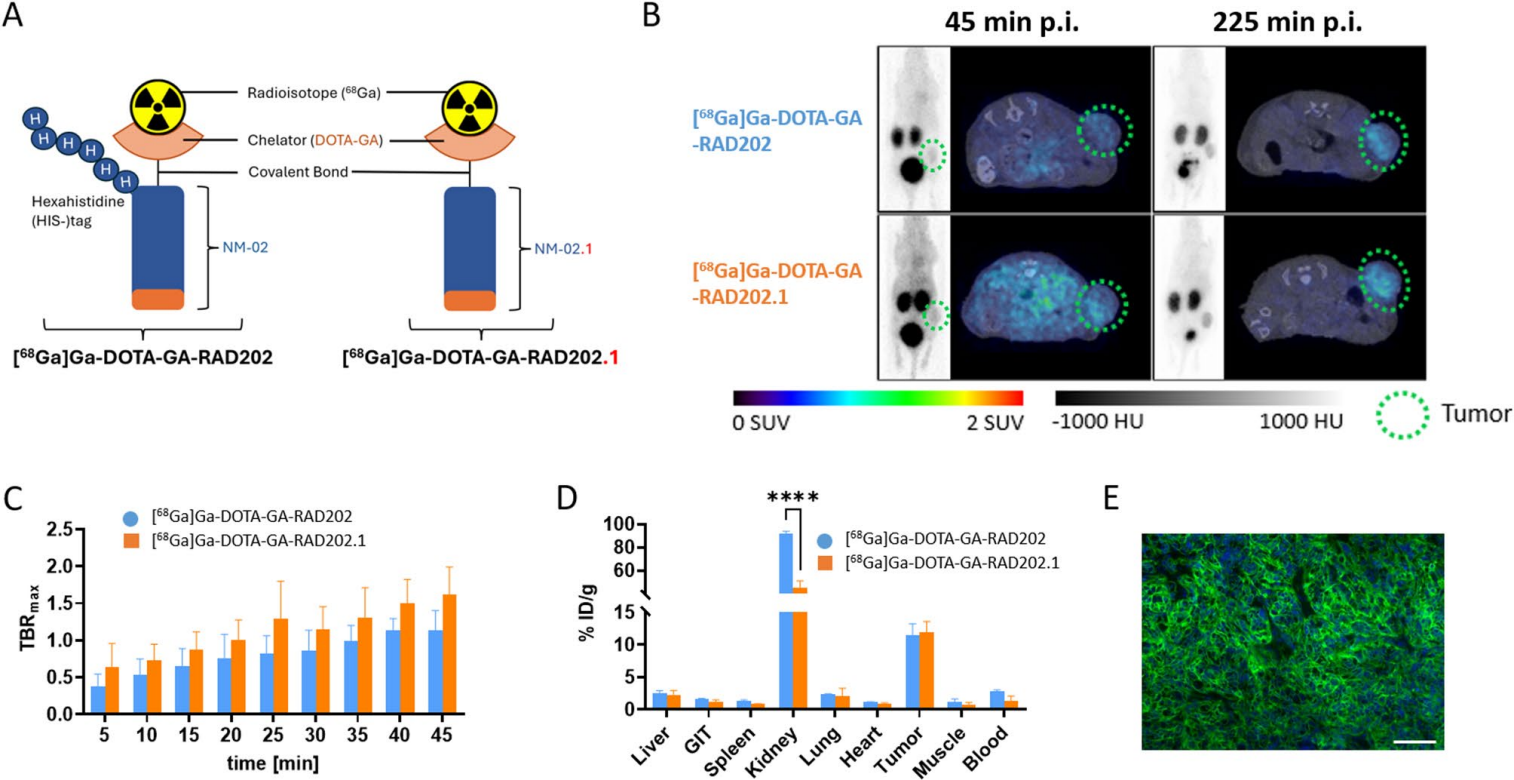
PET/CT imaging of [^68^Ga]Ga-DOTA-GA-RAD202 and [^68^Ga]Ga-DOTA-GA-RAD202.1 in HER2-positive SKOV-3 xenograft model. **(A)** Schematic representation of the products [^68^Ga]Ga-DOTA-GA-RAD202 and [^68^Ga]Ga-DOTA-GA-RAD202.1. **(B)** Representative maximum intensity projections and transaxial PET/CT images at the end of the scan of HER2-positive SKOV-3 xenograft mice injected with [^68^Ga]Ga-DOTA-GA-RAD202 and [^68^Ga]Ga-DOTA-GA-RAD202.1 immediately after injection and 180 min post injection (p.i.). **(C)** The maximal target to blood ratios (TBR_max_) are presented as a function of time. **(D)** Ex vivo analysis after the last PET/CT (4 h p.i.) of the harvested organs are represented as % injected dose (ID)/g tissue. **(E)** Representative fluorescence microscopy image of HER2 staining in a SKOV-3 xenograft section with anti-HER2 (green) and DAPI (blue) for the nuclei. 20x magnification; scale bar: 100 μm. Data in B is presented as mean ± SD (*n* = 3). Significance was tested using two-way ANOVA and Tukey’s post-hoc test (****p ≤ 0.0001; n.s. *p* > 0.05). GIT: gastrointestinal tract.

To assess the specificity and safety of ^68^Ga labeled sdAb-chelators, the products [^68^Ga]Ga-DOTA-GA-RAD202 and [^68^Ga]Ga-DOTA-GA-RAD202.1 (Fig. 1A), were injected in mice bearing SK-BR-3 xenografts, which showed the highest cell uptake in vitro 1 h after tracer application. For intra-individual control, mice received injections with ^68^Ga labeled products on two consecutive days. However, biodistribution was unfavorable, with high uptake in the kidneys and the liver. Additionally, the uptake in the tumor was low and inconsistent. The subsequent staining of tissue sections revealed that the tumor cells, originally HER2-positive in cell culture, were negative for HER2 expression (Figure S4). Therefore, the biodistribution study was continued in the HER2-positive SKOV-3 xenograft model.

The quantitative PET analysis with [^68^Ga]Ga-DOTA-GA-RAD202 in SKOV-3 xenograft model showed moderate to no tracer accumulation in non-target organs, except for the kidney and bladder (Fig. 1B). The SUV_max_ of the tumor, representing tumor uptake, increased in the first 45 min from 0.8 ± 0.1 SUV_max_ at 5 min to 1.2 ± 0.2 SUV_max_. After 225 min it decreased again to 0.7 ± 0.1 SUV_max_ (Figure S5A). The mean uptake obtained for the kidney and liver declined within 225 min (3.8 ± 1.1 SUV_mean_ to 1.3 ± 0.4 SUV_mean,_ and 1.2 ± 0.3 SUV_mean_ to 0.3 ± 0.2 SUV_mean_, respectively) (Figure S5B-C, Table S1).

As the radioactivity in the blood pool decreased over time, the target-to-background ratio (TBR) with blood as background increased over time and reached 1.1 ± 0.2 at 45 min post injection (p.i.) (Fig. 1C). These findings were corroborated by the subsequent ex vivo biodistribution study. The highest [^68^Ga]Ga-DOTA-GA-RAD202 uptake was measured in the kidneys with 91.9 ± 2.7% ID/g, followed by the tumor with 11.5 ± 3.0% ID/g (Fig. 1D). For all other examined organs, the tracer accumulation remained below 3% ID/g.

The PET/CT study with [^68^Ga]Ga-DOTA-GA-RAD202.1 visualized biokinetics profiles similar to those observed for [^68^Ga]Ga-DOTA-GA-RAD202 (Fig. 1A). Accordingly, the excretory organs showed a time-dependent decrease of tracer accumulation (2.9 ± 1.3 SUV_mean_ to 1.0 ± 0.3 SUV_mean_ and 0.9 ± 0.3 SUV_mean_ to 0.1 ± 0.2 SUV_mean_, for kidneys and liver, respectively) (Figure S5). The SUV_max_ analysis for the tumor showed that uptake was similar to [^68^Ga]Ga-DOTA-GA-RAD202 across the various measurement points (Figure S5A). The highest tracer uptake was measured 45 min p.i. with SUV_max_ 1.1 ± 0.3, which decreased to 0.5 ± 0.1 at 225 min p.i. The ex vivo analysis confirmed a similar uptake in the tumor for both tracer (11.5 ± 3.0% ID/g vs. 11.9 ± 2.9% ID/g, for [^68^Ga]Ga-DOTA-GA-RAD202 and [^68^Ga]Ga-DOTA-GA-RAD202.1, respectively). However, in ex vivo analysis the % uptake of [^68^Ga]Ga-DOTA-GA-RAD202.1 in the kidney (44.7 ± 11.0% ID/g vs. 91.9 ± 2.7% ID/g), spleen (0.8 ± 0.1% ID/g vs. 1.4 ± 0.2% ID/g), muscle (0.8 ± 0.5% ID/g vs. 1.1 ± 0.8% ID/g) and blood (1.4 ± 1.3% ID/g vs. 2.8 ± 0.3% ID/g) were lower than those calculated for the HIS-tag conjugate [^68^Ga]Ga-DOTA-GA-RAD202 (Fig. 1C). The declining radioactivity level in the blood led to the time-dependent increase of the TBR, reaching the highest value of 1.6 ± 0.3 at 45 min after the injection of [^68^Ga]Ga-DOTA-GA-RAD202.1 compared with 1.1 ± 0.2 for [^68^Ga]Ga-DOTA-GA-RAD202 at that time. The TBR 225 min after the injection is not displayed, as no activity could be detected in the blood pool at that time (Fig. 1B). However, the mean TBR based on the ex vivo biodistribution study at 225 min p.i. were 4.1 and 8.7 for the [^68^Ga]Ga-DOTA-GA-RAD202 and [^68^Ga]Ga-DOTA-GA-RAD202.1, respectively. The corresponding mean tumor-to-kidney ratios were also 2-fold higher with [^68^Ga]Ga-DOTA-GA-RAD202.1 (0.27) compared with [^68^Ga]Ga-DOTA-GA-RAD202 (0.13).

The subsequent staining with HER2 specific antibody visualized high cell surface expression of HER2 receptor in SKOV-3 xenograft (Fig. 1E).

Tumor specificity was further validated by performing cross-competing or ‘blocking’ experiments, as shown under Supplemental Data (Figure S6 and S7).

### Single cycle vs. fractionated therapy with [^177^Lu]Lu-DOTA-GA-RAD202.1

Synthesis and in vitro studies of [^177^Lu]Lu-DOTA-GA-RAD202.1 can be found in the Supplemental Data (Figure S8). The radiotracer was synthesized with a RCP of 99.8 ± 0.3%.

**Fig. 2:**
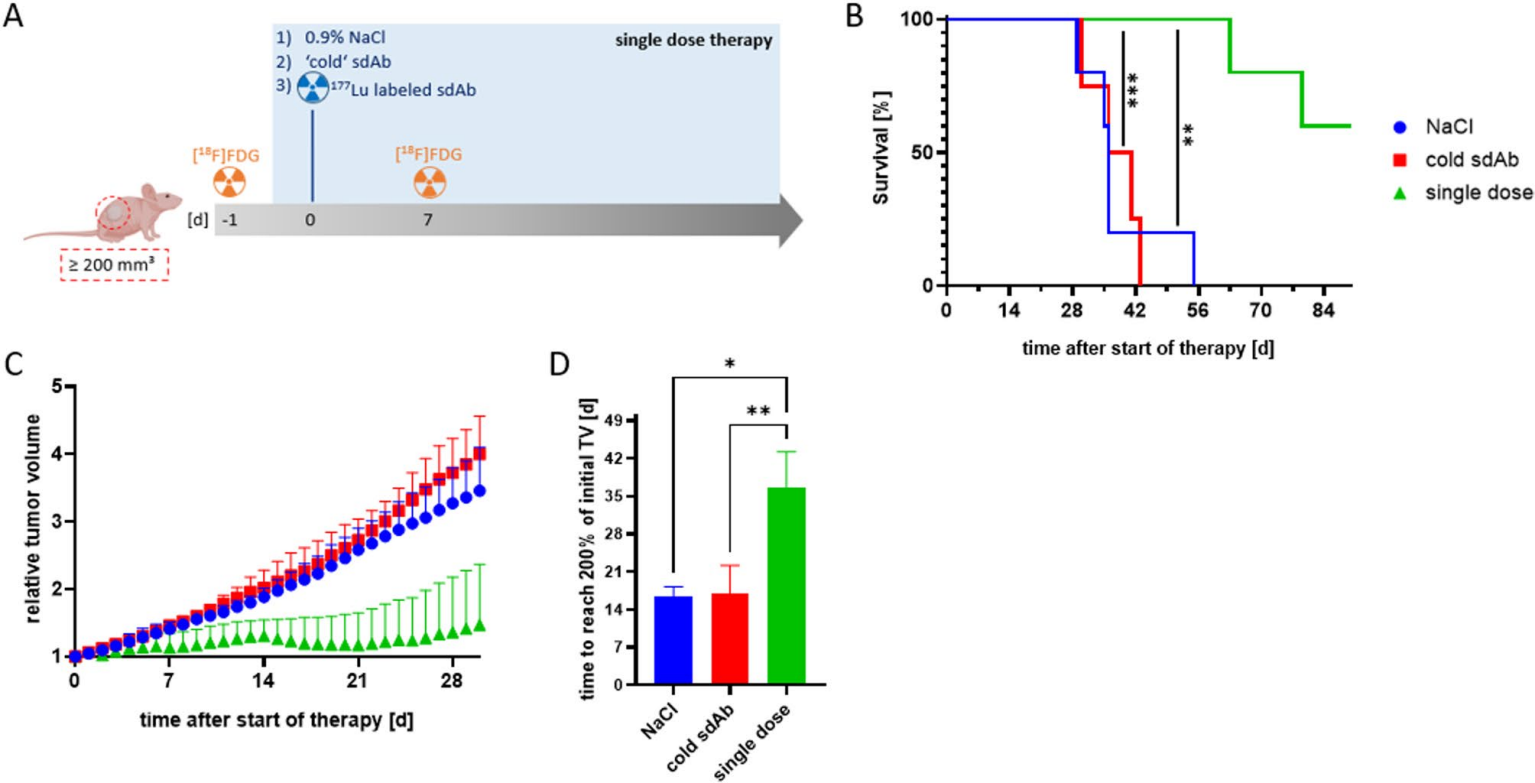
Effect of single cycle therapy with [^177^Lu]Lu-DOTA-GA-RAD202.1. **(A)** Schematic representation of single cycle therapy with [^177^Lu]Lu-DOTA-GA-RAD202.1. Color code in all plots: i.v. application of NaCl (blue), unlabeled ‘cold’ sdAb-chelator conjugate (red) and [^177^Lu]Lu-DOTA-GA-RAD202.1 (green) **(B)** Kaplan-Meier survival analysis for treated mice, where survival was defined as time to reaching a termination criteria. **(C)** The tumor growth curve was obtained through daily measurement after the start of treatment. The arrow indicates the timepoint of therapy injection. **(D)** Time period (in days) until reaching 200% of the initial tumor volume (TV). Significances were test-ed with Welch ANOVA with Brown-Forsythe post-hoc test. (***p ≤ 0.001; ** *p* ≤ 0.01; * *p* ≤ 0.05; n.s. *p* > 0.05). SUV: standard uptake value; TV: tumor volume.

The SKOV-3 xenografted mice were randomized after reaching a tumor volume above 200 mm³ to receive either 54 ± 1 MBq [^177^Lu]Lu-DOTA-GA-RAD202.1 as single dose, NaCl, or the unlabeled sdAb-chelator conjugate DOTA-GA-NM-02.1 (cold sdAb) *via* intravenous injection (Fig. 2A). Animals were finalized upon reaching a humane endpoint for the therapy study (i.e., 15 mm tumor diameter or 1500 mm^3^ tumor volume, exulceration, ≥ 20% weight loss) or at the latest 20 weeks after the start of therapy.

Both, the single cycle and fractionated therapy were well tolerated, which is evidenced by the stable body weights, as the changes in body weight remained below 5%, and the general good condition and behavior of the mice throughout the therapy. Furthermore, Hematoxylin-Eosin (HE) staining on tissue sections of non-target organs did not show any pathological changes (Figure S9).

The single dose treatment group demonstrated the significantly longest median survival of 72 days compared to the groups that were injected with NaCl (38 days) or cold sdAb (38 days) (Fig. 2B). The tumor growth analysis, limited to the first death in the NaCl group, showed that tumor volume 30 days after the therapy had started was significantly smaller for the single dose compared to the two control groups (Fig. 2C). Only control animals reached the tumor volume endpoint of about 1500 mm³. In contrast, only a single dose mouse was killed because of exceeding threshold tumor diameter, and the other four due to the end of observation period, with one showing complete tumor regression. The mean calculated tumor volumes on the day of sacrificing were 693.5 mm³ (single dose), 1246.6 mm³ (NaCl), and 1441.4 mm³ (cold sdAb) (Figure S10). Tumor growth was significantly inhibited in the single dose group, which took longer to reach 200% of initial tumor volume compared to controls (36.5 days vs. 16 and 18 days for NaCl and cold sdAb, respectively) (Fig. 2D).

The combination of the relatively low administered activity and the limited sensitivity of our preclinical SPECT camera precluded the acquisition of high-quality images. Furthermore, the imaging time point of 7 days post-injection would not have yielded SPECT images of sufficient quality, which further restricted the utility of this method. Therefore, the tumor vitality was monitored *via* [^18^F]FDG PET/CT imaging one day before and one week after the treatment (Figure S11). The PET/CT imaging allows visualization and quantification of metabolically active tumor areas and allows earlier and more accurate assessment of treatment effects compared with conventional CT imaging. The mean standardized uptake one week after treatment was lowest in the single dose group, followed by mice injected with cold sdAb and NaCl (0.121, 0.134 and 0.177, respectively), however these differences were not significant. Additionally, no significant changes in SUV_max_ values were observed before and one week after therapy. While the NaCl group showed no trend, SUV_max_ increased in 80% of the mice in cold sdAb group and decreased in 80% of the mice in single dose group.

Histopathology, F4/80 and TUNEL staining of the tumors treated with a single cycle of [^177^Lu]Lu-DOTA-GA-RAD202.1 are shown in Figure S12.

**Fig. 3:**
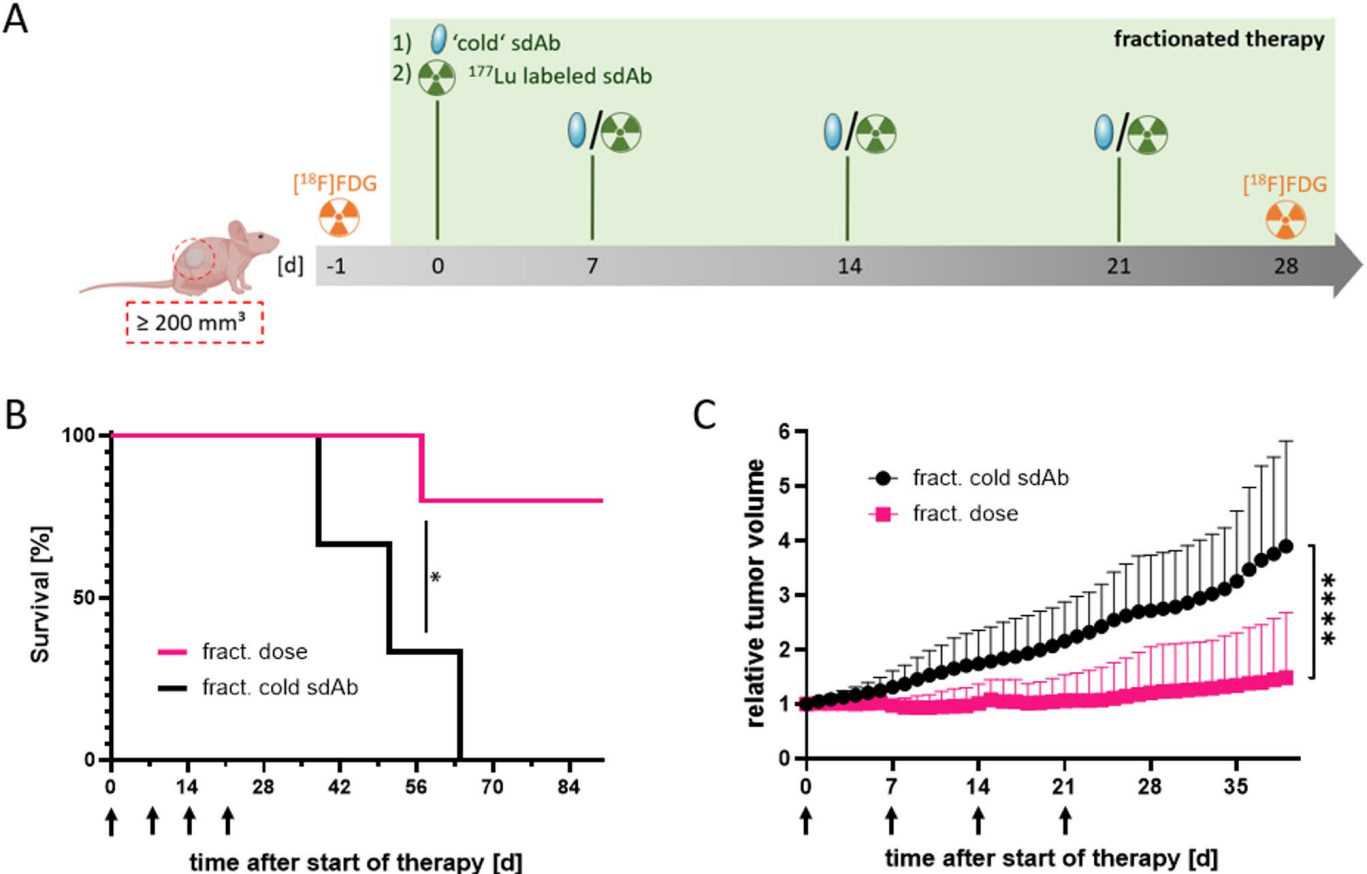
Effect of fractionated therapy with [^177^Lu]Lu-DOTA-GA-RAD202.1. **(A)** Schematic representation of fractionated therapy with [^177^Lu]Lu-DOTA-GA-RAD202.1. Color code in all plots: i.v. application of unlabeled fractionated ‘cold’ sdAb-chelator conjugate (black)and fractionated [^177^Lu]Lu-DOTA-GA-RAD202.1 (pink). **(B)** Kaplan-Meier survival analysis for mice in fractionated therapy group, where survival was defined as time to reaching a termination criterion. Tumor growth curve was obtained through daily measurement after the start of treatment for fractionated therapy until the day of death of the first mouse per group. Data are presented as mean ± SD (*n* ≤ 4). Significances were tested using Welch ANOVA with Brown-Forsythe post-hoc test (B) or unpaired t-test with Welch’s correction (D) (**** *p* ≤ 0.0001; * *p* ≤ 0.05; n.s. > 0.05). The arrows indicate the timepoints of injection.

For fractionated therapy, mice received either four fractions of 13.5 ± 1 MBq [^177^Lu]Lu-DOTA-GA-RAD202.1 each or the unlabeled sdAb-chelator conjugate DOTA-GA-NM-02.1 (cold sdAb) *via* intravenous injection (Fig. 3A).

**Fig. 4:**
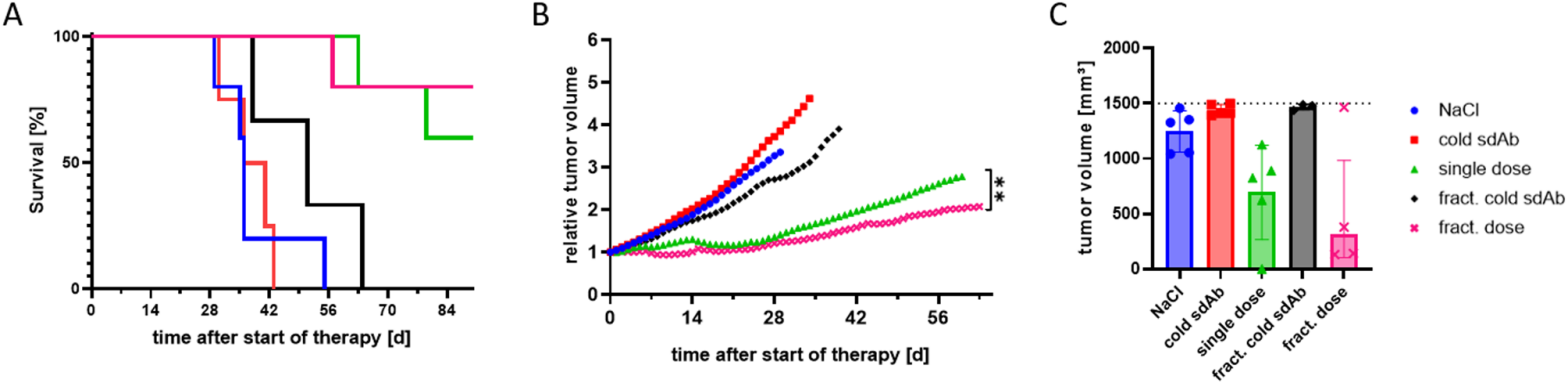
Comparison of the effects of different treatment strategies. (**A**) Kaplan-Meier survival analysis for all therapy groups, where survival was defined as time to reaching a termination criterion. Color code in all plots: i.v. application of NaCl (blue), unlabeled ‘cold’ sdAb-chelator conjugate (red), unlabeled fractionated ‘cold’ sdAb-chelator conjugate (black), single-dose [^177^Lu]Lu-DOTA-GA-RAD202.1 (green) and fractionated [^177^Lu]Lu-DOTA-GA-RAD202.1 (pink). (**B**) Tumor growth curve was obtained through daily measurement after the start of treatment for both therapy groups until the day of death of the first mouse per group. Data are presented as mean ± SD (*n* ≤ 4). Significances were tested using Welch ANOVA with Brown-Forsythe post-hoc test (**A,C**) or unpaired t-test with Welch’s correction (**E**) (**p ≤ 0.01; n.s. > 0.05).

The Kaplan-Meier analysis indicated a median survival of 51 d for the control group (fractionated cold sdAb) and a significantly longer survival with 77 d for the fractionated dose group (Fig. 3B). However, no significant difference in median survival was observed between injection of one dose compared to injection of four doses of [^177^Lu]Lu-DOTA-GA-RAD202.1 (Fig. 4A).

After 30 days, tumor volume was significantly smaller in the fractionated dose group compared to controls (Figs. 3C and 4A). While the control group reached 200% of the initial tumor volume within 25 days, only one mouse in the fractionated group reached this threshold (25 days). A comparison of the relative tumor volumes of the two therapy groups with one and four injections of [^177^Lu]Lu-DOTA-GA-RAD202.1 showed that the fractionated therapy group had a significantly lower tumor volume (Fig. 4B).

The waterfall plot analysis illustrates that all mice in the control group were killed because they reached the end point for tumor volume of about 1500 mm³, while only one mouse from the therapy group was killed due to the same reason. The other three mice from this group were killed due to the end of the observation period of 20 weeks (Figure S13). The mean tumor volumes on the day of killing were 1468.1 mm³ and 529.1 mm³ for cold sdAb and fractionated dose groups, respectively. Statistical analysis revealed significantly smaller tumor volumes at the time of study end for the fractionated dose compared to the control group (Fig. 4C).

The tumor vitality was monitored *via* [^18^F]FDG PET/CT imaging one day before the first injection and one week after the fourth therapy injection (Figure S14). The mean standardized uptake in the fractionated dose group was significantly lower than in the control group (0.169 vs. 0.384, respectively). Comparing SUV_max_ values before therapy and one week after the fourth injection showed no significant differences in either group.

A direct comparison of [^18^F]FDG tumor uptake between single cycle and fractionated therapies is not possible due to different measurement times (7 days vs. 28 days after the first injection). Nevertheless, the metabolic active tumor uptake of the fractionated dose is similar to the values of the single cycle therapy groups, namely NaCl, cold sdAb and single dose (0.177, 0.139 and 0.121, respectively), despite the longer period between the measurement (Figure S15).

Histopathology, F4/80 and TUNEL fluorescence staining of the tumors treated with fractionated [^177^Lu]Lu-DOTA-GA-RAD202.1 are shown in Figure S16.

## Discussion

The primary goal of this study was to evaluate the DOTA-GA conjugated ^68^Ga-labeled sdAb NM-02 (with HIS-tag) and its second-generation variant NM-02.1 (without HIS-tag), now designated as [^68^Ga]Ga-DOTA-GA-RAD202 and [^68^Ga]Ga-DOTA-GA-RAD202.1, respectively, for PET imaging and ^177^Lu-labeled NM-02.1 ([^177^Lu]Lu-DOTA-GA-RAD202.1) for therapy of HER2-positive tumors. The sdAb was successfully conjugated to DTPA or DOTA-GA. The stability with and without HIS-tag on the sdAb was tested at room temperature and at 37 °C in HS. In both cases, the ^68^Ga labeled sdAb without HIS-tag ([^68^Ga]Ga-DOTA-GA-RAD202.1) showed a higher stability. Furthermore, in cellular uptake studies, [^68^Ga]Ga-DOTA-GA-RAD202.1 exhibited a higher affinity for the HER2 receptor after 1 h and 4 h of incubation compared to the HIS-tag conjugate [^68^Ga]Ga-DOTA-GA-RAD202. This is consistent with findings from previous studies showing that the position of the HIS-tag could affect the binding properties. Especially HIS-tags placed at the C-terminus, which is the case for NM-02, can adversely impact binding affinity, while N-terminal HIS-tag may better maintain binding characteristics^7^.

Biodistribution studies with [^68^Ga]Ga-DOTA-GA-RAD202 and [^68^Ga]Ga-DOTA-GA-RAD202.1 showed rapid clearance from non-target organs except for the bladder, and comparable tumor uptake for both radiotracers. Most importantly, the PET imaging-based uptake (SUV_mean_) in off-target organs was substantially lower with [^68^Ga]Ga-DOTA-GA-RAD202.1 compared to [^68^Ga]Ga-DOTA-GA-RAD202 with similar tumor uptake resulting in higher tumor-to-organ ratios with the former. This was corroborated in the ex vivo biodistribution study showing that the %uptake in the kidneys, spleen, muscle and blood was significantly lower with [^68^Ga]Ga-DOTA-GA-RAD202.1 compared with [^68^Ga]Ga-DOTA-GA-RAD202 (44.7 ± 11.0% ID/g vs. 91.9 ± 1.6% ID/g for the kidneys) but similar tumor uptake, again resulting in higher uptake ratios for these organs with a less pronounced difference in the tumor-to-liver ratio. In fact, the mean tumor-to-blood and tumor-to-kidney ratios were found to be more than 2-fold higher with [^68^Ga]Ga-DOTA-GA-RAD202.1 compared with [^68^Ga]Ga-DOTA-GA-RAD202. The lower kidney uptake with the sdAb without as opposed to with HIS-tag has also been previously shown by Xavier et al. using the HER2 targeting sdAb 2Rs15d radiolabeled with ^68^Ga *via* NOTA derivative for HER2 PET imaging in SKOV-3 xenograft mice^8^. Taken together, these findings suggest that [^68^Ga]Ga-DOTA-GA-RAD202.1 is clearly superior for PET imaging of HER2-positive tumors. Since the higher tumor-to-organ ratios translate into higher therapeutic index, it is only logical that [^177^Lu]Lu-DOTA-GA-RAD202.1 and not [^177^Lu]Lu-DOTA-GA-RAD202 was chosen for the mice therapy studies and should be the candidate of choice for future clinical studies. The lower kidney uptake with the ^177^Lu-RAD202.1 is critical to reducing potential nephrotoxicity from this treatment.

In the biodistribution studies by Xavier et al., the ex vivo analysis of the harvested organs 225 min after the injection showed a tumor uptake of 0.1 ± 0.03% ID and a kidney uptake of 6.3 ± 1.1% ID for the sdAb without HIS-tag^8^. Despite the same study design, the uptake values with [^68^Ga]Ga-DOTA-GA-RAD202 and [^68^Ga]Ga-DOTA-GA-RAD202.1 are higher for tumor (1.6 ± 0.6% ID and 2.1 ± 0.2% ID, which corresponds to 11.5 ± 3.0% ID/g and 11.9 ± 2.9% ID/g, respectively), and kidney (14.1 ± 0.3% ID and 7.6 ± 1.1% ID, which corresponds to 91.9 ± 2.7% ID/g and 44.7 ± 11.0% ID/g, respectively) and the tumor-to-kidney ratios are also higher. This could indicate a higher stability of our tracers in vivo.

Specific tumor uptake was demonstrated by a blocking study, as the uptake of the radioactive product in the ex vivo analysis decreased by 55.5% for [^68^Ga]Ga-DOTA-GA-RAD202 and by 59.2% for [^68^Ga]Ga-DOTA-GA-RAD202.1.

SKOV-3 xenografted mice were used to investigate the therapeutic efficacy of a single dose [^177^Lu]Lu-DOTA-GA-RAD202.1 compared to a fractionated dosing. Both, the single cycle and fractionated therapies were well tolerated, which is evident by the stable body weight and normal morphology of non-target tissues. Analysis of tumor volume, tumor growth inhibition, and overall survival of the mice indicated the efficacy of both therapeutic strategies. The fractionated therapy demonstrated even greater effectiveness in inhibiting tumor growth compared to the single dose. In contrast to the single dose, mice did not reach 200% of the initial tumor volume until the end of the observation period and had a significantly lower tumor volume throughout. Although the group size in the fractionated therapy arm was deliberately limited (*n* = 3–4), the observed treatment effects remained statistically significant. This decision was ethically motivated and follows the 3R principles (Replace, Reduce, Refine), aiming to reduce animal numbers while preserving scientific validity. The enhanced outcome might be explained by the application of fractionated doses targeting tumor cells in different cell cycle phases. Cells in the G1 phase are less radiosensitive and least sensitive in the latter part of the S phase. Most radiosensitive cells are in G2-M phase^9,10^. Since tumor cells within a lesion proliferate asynchronously, subpopulations are distributed across different phases of the cell cycle at any given time. Although the weekly dosing interval is not aligned with the short duration of the cell cycle itself, repeated administrations increase the probability of targeting a larger proportion of cells during radiosensitive phases over the course of therapy.

HE staining of tumor tissue and the absence of [^18^F]FDG uptake in the core region of the tumors revealed a central necrosis formation, which was demonstrated for different HER2-positive xenograft models in several studies before^11–14^. These necrotic areas are infiltrated by macrophages, which are specialized phagocytes that recognize, engulf and digest microbes, dying cells and cellular debris, cancer cells and foreign bodies, thus clearing tissues from damaged cells and microorganisms. F4/80 is a plasma membrane glycoprotein that is frequently used as a macrophage marker in mice. The fluorescence staining with F4/80 showed that the perinecrotic regions indicated by HE coincidded with the F4/80-positive areas. Control groups showed more macrophages and leukocytes due to larger tumors and necrotic areas. TUNEL staining was used to detect apoptotic cells by labeling the DNA fragments generated by the endogenous radiation. For both therapy regimes, a high number of apoptotic cells were detected in the viable outer tumor edges.

The results obtained with [^177^Lu]Lu-DOTA-GA-RAD202.1 are in line with the previously reported therapeutic effects of other HER2-targeted sdAbs. For example, iso-^211^At-SAGMB-anti-HER2-sdAb conjugates have been shown to significantly delay tumor growth and prolong survival in a mouse model of HER2-expressing breast cancer with no apparent toxicity to normal tissue^15^. The prolongation in median survival was 495% with iso-^211^At-SAGMB-5F7 and 414% with iso-^211^At-SAGMB-VHH_1028. Injection of [^177^Lu]Lu-DOTA-GA-RAD202.1 prolonged median survival by 89.5% in the single dose group and by 51% in the fractionated dose group compared to the respective control group with unlabeled ‘cold’ sdAb-chelator conjugates. Different length of the observation period (84 days vs. 196 days), study design and type of radiation (beta vs. alpha emitter) might explain the difference in the evaluated therapeutic efficacies. Another major difference is the starting tumor volume for the therapy. Feng et al.^15^. started the therapy with tumors of 120 mm³ and D’Huyvetter et al.^16^. with tumor volumes of 20 to 30 mm³, compared to ≥ 200 mm³ in the present study. Since it is known that tumor size significantly influences the effectiveness of various therapeutic procedures such as surgery, chemotherapy and radiation^17,18^, the tumor growth regression in 20% of the animals in our study highlights the therapeutic potential of [^177^Lu]Lu-DOTA-GA-RAD202.1.

Finally, the lower prolongation of median survival in the fractionated compared with the single dose group despite similar median survivals in both groups (77 vs. 71 days, respectively) is due to the longer median survival in the fractionated compared with the single dose cold sdAb (51 vs. 38 days, respectively). One possible explanation is that the fractionated cold sdAb may have been somewhat effective, and more effective than the single dose cold sdAb at least initially. Indeed, at 30 and 33 days posttherapy, the mean relative tumor volume was noticeably lower with the former compared with the latter (Fig. 4B). However, this effect was apparently short lived as the mean tumor at the day of killing was similar with single and fractionated cold sdAb. Importantly, the greater tumor growth inhibition with the fractionated compared with the single dose [^177^Lu]Lu-DOTA-GA-RAD202.1 was not due to lower initial tumor volumes in both treatment groups (228.7 mm³ vs. 227.4 mm^3^, respectively).

## Conclusion

This study demonstrated that the HER2-targeted sdAb [^68^Ga]Ga-DOTA-GA-RAD202.1 without HIS-tag is superior for PET imaging of HER2-positive tumors compared with the sdAb [^68^Ga]Ga-DOTA-GA-RAD202 with HIS-tag due to the higher tumor-to-organ ratios with the former. [^68^Ga]Ga-DOTA-GA-RAD202.1 also showed higher stability in vitro and higher affinity for the HER2 receptor. The [^177^Lu]Lu-DOTA-GA-RAD202.1 therapy was well tolerated, as single as well as fractionated dose. However, fractionated dosing proved more effective in inhibiting tumor growth compared to a single dose regimen. For this reason, [^177^Lu]Lu-DOTA-GA-RAD202.1 is considered to be a good candidate for translation into human application and is currently in clinical development by Radiopharm Theranostics.

## Methods

### Preparation of and in vitro studies with [^68^Ga]Ga-DOTA-GA-RAD202, [^68^Ga]Ga-DOTA-GA-RAD202.1 and [^177^Lu]Lu-DOTA-GA-RAD202.1

The methods pertaining to radiopharmaceutical preparation and in vitro studies with [^68^Ga]Ga-DOTA-GA-RAD202, [^68^Ga]Ga-DOTA-GA-RAD202.1 and [^177^Lu]Lu-DOTA-GA-RAD202.1 using the three HER2-positive cell lines (BT474, SK-BR-3 and SKOV-3) and the one HER2-negative cell line (MDA-MB231) are described in the Supplemental Data.

### Biodistribution studies with ^68^Ga-RAD202 and ^68^Ga-RAD202.1

All animal experiments were approved by a German competent authority (Landesamt für Natur, Umwelt und Verbraucherschutz Nordrhein-Westfalen) for compliance with the Animal Protection Act, in conjunction with the regulation for the protection of animals used for experimental and other scientific purposes.

Animal housing and tumor cell inoculation was performed as described previously^19^. Mice (n = 6) inoculated with HER2-positive cell line were injected with 10 MBq of the ^68^Ga-labeled sdAbs, three with [^68^Ga]Ga-DOTA-GA-RAD202 (approximately 20 µg NM-02) and three with the [^68^Ga]Ga-DOTA-GA-RAD202.1 (approximately 20 µg NM-02.1), alongside with 50 µL Gelofusine^®^ (40 mg/mL) when tumors reached a volume > 200 mm³. The mice injected with either sdAb were randomly selected. The following day (at least after 10 half-lives of ^68^Ga = 68 min) the groups of three mice each were injected with a different ^68^Ga-labeled sdAb (either [^68^Ga]Ga-DOTA-GA-RAD202 or [^68^Ga]Ga-DOTA-GA-RAD202.1 depending on which sdAb was injected on the first day). Following each injection, the first dynamic PET (45 min) scan was performed with the setting described previously immediately after the injection^19^ with a second PET/CT scan following 180 min later. After the last scan, with identical scan sequence done the day before, mice were killed, and organs were collected and measured in the gamma counter. For the inhibition study (n = 6), mice received an additional injection of 200 µg of unlabeled, ‘cold’ sdAb together with each ^68^Ga-labeled sdAbs. The experimental procedure was identical. Post imaging procedures were performed as described before^19^. Standard uptake values (SUVs) were determined by hand-drawing volumes of interest (VOIs) using CT anatomical guidelines.

### Therapy study with [^177^Lu]Lu-DOTA-GA-RAD202.1

The therapeutic effect of a single cycle therapy was evaluated in 15 mice when tumors reached a volume > 200 mm³ (*n* = 5 [^177^Lu]Lu-DOTA-GA-RAD202.1; *n* = 5 0.9% NaCl (NaCl), *n* = 5 DOTA-GA-NM-02.1). Under isoflurane anesthesia, mice were injected with [^18^F]FDG (5 MBq in 25 µL, Advanced Accelerator Applications) and scanned immediately in the PET scanner for 30 min, followed by a 3-min CT scan. The next day, mice received 50 µL Gelofusine^®^ (40 mg/mL) and 54 ± 1 MBq [^177^Lu]Lu-DOTA-GA-RAD202.1 (~ 100 µg NM-02.1), NaCl, or 100 µg NM-02.1-DOTA-GA intravenously. The [^18^F]FDG scan was repeated one week after the beginning of the therapy.

The effect of fractionated therapy was evaluated in a total of 7 mice (*n* = 4 [^177^Lu]Lu-DOTA-GA-RAD202.1 and *n* = 3 for NM-02.1-DOTA-GA). Under isoflurane anesthesia, mice were injected with [^18^F]FDG (5 MBq in 25 µL) and scanned in the PET scanner for 30 min, followed by a 3-min CT scan. The next day, the mice were injected intravenously with 50 µL Gelofusine^®^ (40 mg/mL) and a quarter of the single dose for [^177^Lu]Lu-DOTA-GA-RAD202.1 (approximately 13.5 ± 1 MBq; 25 µg NM-02.1) or 25 µg NM-02.1-DOTA-GA was injected for four weekly injections. The [^18^F]FDG Scan (5 MBq in 25 µL) was performed for all mice one week after the fourth injection.

After sacrificing the mice, the harvested organs were examined using Hematoxylin–Eosin-, F4/80 immunofluorescence and TUNEL fluorescence staining.

## Supplementary Information

Below is the link to the electronic supplementary material.

Supplementary Material 1

## Data Availability

All data generated or analyzed during this study are available from the corresponding author.
