# Supplementary material for: Theranostic application of radiolabeled HER2-targeting single-domain antibodies (RAD202) in preclinical setting
Source: Sci Rep. 2025 Nov 4;15:38468. doi: 10.1038/s41598-025-25679-w (PMC12586553; doi:10.1038/s41598-025-25679-w)
Supplement: Supplementary file 1 — Supplementary Material 1 [file 41598_2025_25679_MOESM1_ESM.docx]

**Supplementary Data**

**Methods**

**Purification of the single-domain antibody**

To remove metal traces, 10 μL of 50 mM EDTA in PBS was added to 500 μL of sdAb stock (2 mg/mL) and incubated for 30 min at room temperature (25 °C). The mixture was then loaded onto a conditioned PD MiniTrap™ G-25 column, eluted with 1.2 mL carbonate-bicarbonate buffer (0.05 M, pH 9.5), and collected. Protein recovery was measured *via* HPLC using a calibration curve.

**Chelator Conjugation to single-domain antibodies**

Purified NM-02 (with HIS-tag) or NM-02.1 (without HIS-tag) was mixed with different amounts of reactive chelator solutions (10 mM in carbonate-bicarbonate buffer at pH 9.5) of p-SCN-Bz-DOTA-GA (DOTA-GA, CheMatech) or p-SCN-Bn-CHX”-DTPA (DTPA, Macrocyclics) to create sdAb-chelator conjugates in different loading ratios.

| sdAb-to-chelator ratio | Chelator solution per mg of sdAb [µL] |
| --- | --- |
| 1/10 | 71 |
| 1/20 | 141 |
| 1/40 | 282 |

After 16 h of incubation in the dark at room temperature (25 °C), the mixture was diluted with PBS to 2.5 mL and transferred to a PD-10 column. Elution with 3.5 mL PBS collected the sdAb-chelator conjugate into a 10 kDa Amicon® Ultra-4 filter. The solution was centrifuged at 4000 RPM for 5 min, until 50–100 μL remained, rinsed with 100 μL PBS and diluted to 500 μL. Purity and concentration were confirmed by HPLC and the bicinchoninic acid (BCA) assay.

**Preparation of [^68^Ga]Ga-DOTA-GA-RAD202 and** **[^68^Ga]Ga-DOTA-GA-RAD202.1**

Ammonium acetate buffer (3 M, pH 7) was added to acidic (0.1 M HCl) ^68^Ga^3+^ solution (250 µL, 160 – 220 MBq, NRF-iThemba LABS) to adjust the pH to 4. The sdAb chelator conjugate DTPA‑NM‑02 (100 µL, 1 mg/mL, sdAb-to-chelator ratio 1/20) was added, mixed and diluted to 500 µL with ultrapure water and kept at room temperature (25°C) for 25 min. The labeling reactions with DOTA-GA-NM-02 (100 μL, 1 mg/mL, sdAb-to-chelator ratio 1/20) was performed at 55 °C for 30 min at pH 4. The radiolabeled NM-02 ([^68^Ga]Ga-DOTA-GA-RAD202) or NM-02.1 ([^68^Ga]Ga-DOTA-GA-RAD202) was purified twice using Amicon® Ultra-0.5 centrifugal units. Samples were analyzed by HPLC.

**Preparation of [^177^Lu]Lu-DOTA-GA-RAD202.1**

Ammonium acetate buffer (0.5 M, pH 7) was added to HCl (0.6 M) to adjust the pH to 4, then ^177^Lu^3+^ (70 - 80 MBq in 0.04 M HCl, Monrol) was added. The sdAb-chelator conjugate DOTA-GA-NM-02.1 (100 μL, 1 mg/mL) was added, and the mixture was briefly mixed and heated at 55 °C. After 30 min, the product ^177^Lu-labeled NM-02.1 ([^177^Lu]Lu-DOTA-GA-RAD202.1) was purified twice using Amicon® Ultra-0.5 centrifugal units and analyzed by HPLC and TLC for radiochemical conversion (RCC).

**Chromatographic analysis**

The HPLC consists of a Smartline Pump 1000, the UV detector 2500 (wavelength set at 280 nm), the radioactivity detector Gina Star and the software Gina Star 5.9. Size exclusion chromatography (SEC) was performed on a BioSep SEC-s2000 column, and a isocratic mobile phase of 50% acetonitrile and 50% aqueous eluent (0.1% trifluoroacetic acid, 2% citric acid in 0.9% NaCl) at a flow rate of 2 mL/min for 18 min. Thin layer chromatography (TLC) was performed on silicic acid impregnated chromatographic sheets using citrate buffer pH 5.0 as the eluent.

***In vitro* stability of radiolabeled products**

Shelf life was tested by storing the product at room temperature (25 °C) for up to 140 h. Stability was tested in PBS and human serum (HS) at two dilution ratios (1/1 and 1/9). The products (2 MBq) were diluted in PBS or HS and incubated at 37 °C. Samples were taken and analyzed by TLC to determine radiochemical purity (RCP).

**Cell culture**

The HER2-negative breast cancer cell line MDA-MB231 and HER2-positive breast cancer cell line SK-BR‑3 were obtained from American Type Culture Collection and Cell Line Service, respectively. Both were cultured in Dulbecco’s Modified Eagle’s Medium (DMEM) (Pan Biotech), supplemented with 10% fetal bovine serum. HER2-expressing breast cancer cell line BT474 was purchased from Cell Line Service and cultured in Roswell Park Memorial Institute media (RPMI1640) (Pan Biotech) enriched with 20% fetal bovine serum. HER2-positive ovarian cancer cell line SKOV-3 was purchased from American Type Culture Collection and grown in DMEM/F12 growth medium (Thermo Fisher Scientific) supplemented with 20% fetal bovine serum. All media were supplemented with 1% penicillin/streptomycin. All cells were tested biweekly for mycoplasma contamination.

**Cell binding**

The cellular uptake of radiolabeled sdAbs was studied in BT474, SK-BR-3, SKOV-3 and MDA‑MB231 cells. Cells were seeded 24 h before the experiment (1.0*10⁵ cells/well) and incubated with radioactive sdAbs (0.5 MBq/well) for 1 h and 4 h at 37 °C and 5% CO_2_. Inhibition studies included a co-incubation with 100-fold excess of unlabeled HER2-targeting sdAb. After incubation, media was removed, cells were washed, harvested with trypsin, and measured in a Wizard2 gamma counter (PerkinElmer). The decay corrected values were normalized to total protein concentration per well determined by a BCA assay to give final values in percent activity per mg of protein.

**Cell viability and proliferation XTT assay**

Cells (5*10³ per well) were plated in a 96-well plate and incubated for 24 h. Lutetium-177 labeled sdAbs (0-5 MBq in 100 μL) were added and incubated for 4 h at 37 °C and 5% CO_2_. After removing the radioactive medium, cells were washed with PBS and incubated for 72 h. Fresh medium and XTT solution (70 μL, Thermo Fisher Scientific) were added, followed by a 2 h incubation. Absorbance was measured at 450 nm and 660 nm using a microplate reader (Tecan).

**Tumor model and animal care**

All animal experiments were approved by a German competent authority (Landesamt für Natur, Umwelt und Verbraucherschutz Nordrhein-Westfalen) for compliance with the Animal protection Act, in conjunction with the regulation for the protection of animals used for experimental and other scientific purposes (file number 81-02.04.2020.A138).

Female Rj:ATHYM-Foxn1nu/nu mice at 6-8 weeks of age from Janvier labs were used for developing subcutaneous HER2-positive tumor. Animals were housed in filter top cages at a constant temperature of 20-25°C, 45-65% relative humidity with a 12-h-light/12-h-dark cycle and had free access to food and water.

For tumor implantation, 5 × 10^6^ SK-BR-3 or SKOV-3 cells were suspended in a 1:1 (volume) mixture of culture media and Matrigel® matrix. Mice were inoculated with this cell suspension (max 200 μl) subcutaneously in the right flank. Tumor growth was monitored daily using calliper measurements. Tumor volume was calculated daily according to the formula:

Tumor volume=Tumor Length× (Tumor breadth)^2^× 0,52

Upon reaching a tumor volume of more than 200 mm^3^, the animals were used for experiments.

**Image processing**

The reconstructed PET/CT data was exported and post-processed in PMOD software.

To calculate the blood pool background activity, a volume of interest (VOI), comprising of ~10 consecutive slices, was manually placed above the heart. The mean activity (in kBq/cc) and the average of the top five hottest voxels were recorded from this VOI. Afterwards, an automatic isocontour was generated using 20 kBq/cc as the minimal threshold for the images generated 3 h post injection. The threshold value for the images immediately after injection was applied decay corrected (126.5 kBq/cc).

The same technique was applied for the VOIs of the kidney, liver and tumor. Upon completion of all target VOIs, the mean counts were recorded. the mean activity (in kBq/cc) and the hot average was recorded.

The mean standard uptake value (SUVmean) was calculated using the following formula:

SUVmean=mean activity/ decay corrected ID×𝑚𝑚𝑜𝑢𝑠𝑒

where mean activity is the mean activity in the target (in kBq/cc), ID is the injected dose (in kBq) injected in the respective mouse and m_mouse_ is the weight of the mouse (in g).

The maximal standard uptake value (SUVmax) was calculated using the following formula:

SUVmax=hot average/decay corrected ID×mmouse

where hot average is the average of the top five hottest voxels in the target (in kBq/cc), ID is the injected dose (in kBq) injected in the respective mouse and m_mouse_ is the weight of the mouse (in g).

The maximal tumor to background ratio was calculated by the ratio of hot averages from blood pool to tumor.

To calculate the total metabolic active tumor uptake a 5 mm sphere was initially placed in the center point of the tumor and manually adjusted to fit the tumor contour based on the CT. Afterwards, an automatic isocontour was generated using a minimal SUV threshold of 2. The total activity (in kBq/cc*ccm) and the aver-age of the top 5 hottest voxels (hot average) were recorded from this newly generated VOI.

In order to quantify the PET data and to correct for the injected dose and the weight of the animal, the FDG total activity of the tumor was calculated using the following formula:

metabolic active tumor uptake=(total activity/ ID)×m_mouse_

where total activity is the total activity (in kBq/cc*ccm) of the metabolic active tu-mor VOI, ID is the injected dose (in kBq) injected in the respective mouse and m_mouse_ is the weight of the mouse (in g).

**Haematoxylin–Eosin staining**

Cryopreserved slides (5 μm) were thawed at room temperature for 10 min, washed in water for 5 min, stained with hematoxylin (10 min), and rinsed again (5 min). Sections were incubated with 1% eosin (1 min), washed twice in PBS (5 min each), dehydrated in graded ethanol (70%, 96%, 100%) and xylene, then mounted with Pertex.

**F4/80 immunofluorescence staining**

To identify macrophages within the tumor samples, cryopreserved slides (5 μm) were thawed for 10 min, washed thrice in PBS (5 min each), stained with F4/80 antibody (1:250 in 10% goat serum/PBS, Abcam) for 1 h at room temperature, washed, and incubated with Alexa Fluor488-conjugated secondary antibody (1:500 in 10% goat serum/PBS, Cell Signalling) for 1 h at room temperature. Slides were then mounted with ROTI®Mount FluorCare DAPI.

**TUNEL fluorescence staining**

To identify apoptotic cells within the tumor samples, cryopreserved slides (5 μm) were thawed for 10 min, fixed in 4% paraformaldehyde (20 min), washed in PBS (30 min), and permeabilized with 0.1% Triton X-100 (2 min on ice). TUNEL (Terminal deoxynucleotidyl transferase-mediated dUTP Nick End Labeling) reaction mix (Sigma-Aldrich) was applied for 1 h at 37 °C, followed by three PBS washes (5 min each). Slides were then mounted with ROTI®Mount FluorCare DAPI.

**Results**

**Preparing single domain antibody-chelator conjugates**

The HER2-targeting sdAbs were purified using a PD MiniTrap™ G-25 column to remove protein aggregates. Representative chromatograms show the purity of the filtered fractions (Figure S1).


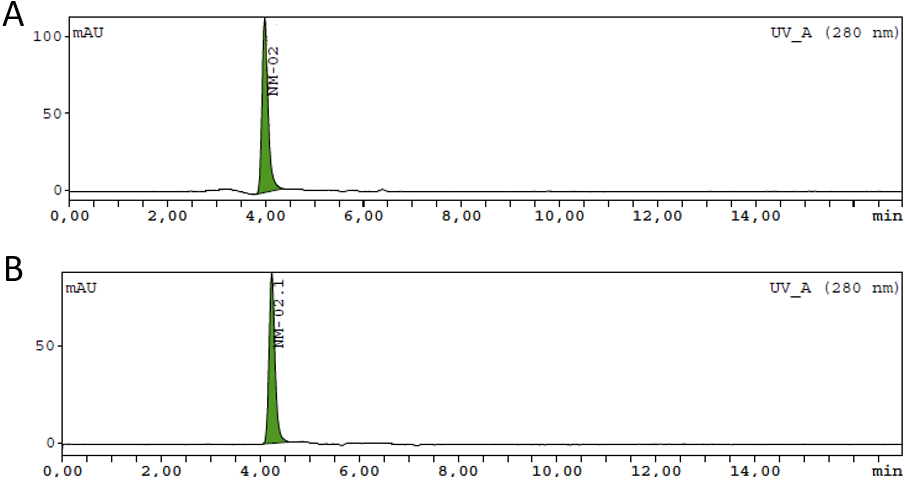


**Figure S1:** **HPLC analysis of the purified single domain antibody NM-02 and NM-02.1**

Chromatograms after injection of 15 μL purified protein (2 mg/mL), **(A)** NM-02 and **(B)** NM-02.1. The absorbance unit (mAU) at λ = 280 nm is plotted against the retention time (t_r_). Flow rate: 2 mL/min, column: BioSep SEC‑s2000 column (300 x 7.8 mm), eluent: 50% acetonitrile and 50% aqueous eluent (0.1% trifluoroacetic acid, 2% citric acid, 0.9% NaCl)

Purity of the sdAb-chelator conjugates was also analyzed by HPLC. There were no identifiable impurities found at 280 nm for any of the conjugates (chemical purity = 99.9%). The retention time (t_r_) in the UV channel matched the t_r_ of the unconjugated sdAbs.

The labeling reactions of the chelator DTPA with ^68^Ga and ^177^Lu have been previously described [1]. With the DOTA-GA-NM-02 conjugate (Figure S2A-B) the temperature, time and pH during radiolabelling with ^68^Ga were adjusted to achieve a higher RCC. Also, the derivatization degree of sdAb-chelator conjugates was optimized and the radiolabeling kinetics were investigated to achieve the highest RCC (Figure S2C-D) and specific uptake in HER2-positive cells (Figure S2E-F).

The lower cell uptake observed for the DTPA conjugates compared to DOTA-based conjugates can be explained by differences in the chemical and biological properties of the two chelators. DOTA forms thermodynamically and kinetically more stable complexes with radiometals such as ^68^Ga and ^177^Lu than DTPA does, thereby reducing the likelihood of partial dechelation, i.e. loss of radiometal. In addition, the overall charge and hydrophilicity of DTPA-conjugates is higher, which may negatively affect membrane interaction and target binding.


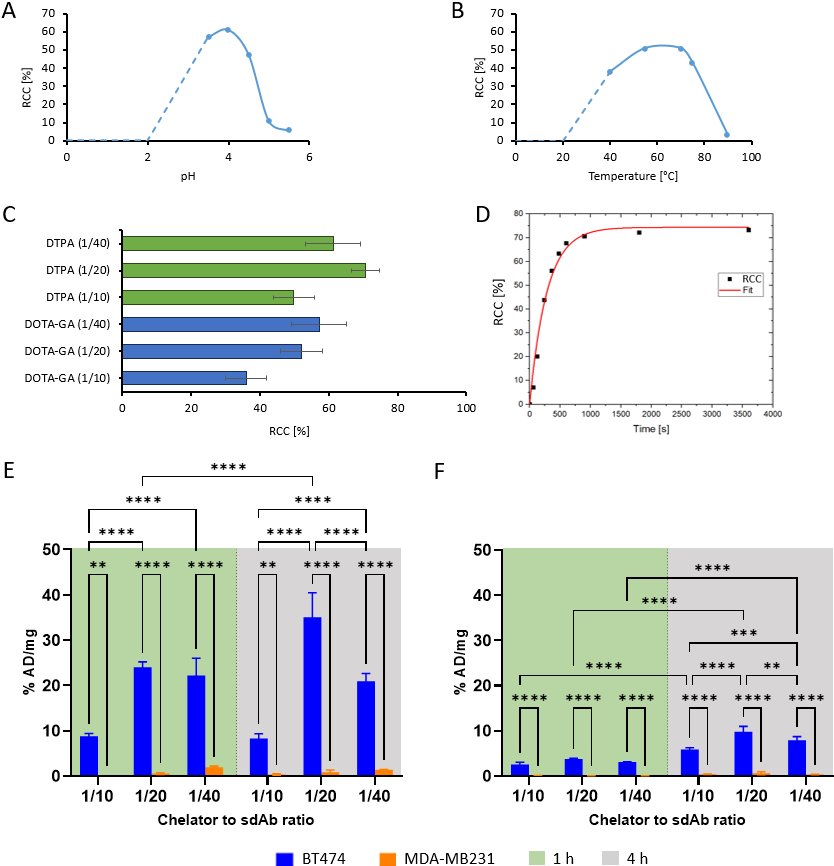


**Figure S2: Optimization of radiolabeling conditions with ^68^Ga, radiochemical conversion and cell uptake of sdAb-chelator conjugates obtained under different derivatization ratios (sdAb:chelator = 1/10, 1/20, 1/40)**

**(A)** Radiochemical conversion (RCC) of DOTA-GA-NM-02 (1/20 derivatization ratio) with ^68^Ga determined by HPLC is shown in dependence of pH with reaction conditions: T = 70°C, *c_protein_* ≈ 10 μM, *t* = 15 min. **(B)** The RCC determined by HPLC is shown in dependence of temperature with reaction conditions: pH 4, *c_protein_* ≈ 10 μM, *t* = 15 min. The dotted line is the assumed course in continuation of the fitted line and based on experience. RCC: radiochemical conversion. **(C)** Reaction conditions: pH 4, *T* = 70 °C for DOTA-GA and *T* = 25 °C for DTPA, *c_p_*_rotein_ ≈ 10 μM, labeling time *t =*10 min. Data are presented as mean ± SD (n = 3). (D) Radiolabeling kinetics of ^68^Ga labeled DOTA-GA-NM-02 at pH4, T = 55°C **(E-F)** Cell uptake of different derivatization degrees of single domain antibody (sdAb) with chelator conjugates of ^68^Ga labeled **(E)** DOTA-GA-NM-02 and **(F)** DTPA-NM-02 after 1 h and 4 h as percent uptake of applied dose (AD) per mg protein in HER2 positive (BT474) and HER2 negative cells (MDA-MB231). Data are presented as mean ± SD (n = 3). Significance was tested using two-way ANOVA with Tukey’s post-hoc test (**** p ≤ 0.0001; *** p ≤ 0.001; ** p ≤ 0.01; n.s. p > 0.05). AD: applied dose; RCC: radiochemical conversion; sdAb: single domain antibody.

**Synthesis and stability studies with ^68^Ga**

The purified sdAb-chelators (DOTA-GA) with 1/20 derivatization ratio were radiolabeled at pH 4 and 55 °C for 30 min with ^68^Ga. The overall radiochemical yield (RCY) was 58.9 ± 10.8% and 40.4 ± 22.3% for the sdAb-chelators without HIS-tag and with HIS-tag, respectively. The t_r_ were 4.18 min for [^68^Ga]Ga-DOTA-GA-RAD202 and t_r_ = 4.48 min for [^68^Ga]Ga-DOTA-GA-RAD202.1 (Figure S3D-E), whereas the higher t_r_ of 5.43 min is assigned to protein-free ^68^Ga-labeled DOTA-GA and t_r_ of 5.85 min to free ^68^Ga (Figure S3A-B).

The stability of radiolabeled sdAb-chelators with and without HIS-tag was tested in saline at 25 °C and in PBS or HS at 37 °C. Figure S3F shows that both products retained >99% RCP for 2 h in saline at 25 °C. After 4 h, [^68^Ga]Ga-DOTA-GA-RAD202.1 showed higher stability than [^68^Ga]Ga-DOTA-GA-RAD202 (98.8 ± 1.5% vs. 97.9 ± 1.7%). For [^68^Ga]Ga-DOTA-GA-RAD202, stability varied depending on the medium (Figure S3G). While the product maintained a purity of over 97.6 ± 2.0% in HS 1/1 for up to 4 h, it decreased to 94.7 ± 3.5% in HS 1/9. In PBS, [^68^Ga]Ga-DOTA-GA-RAD202 had a purity of 95.3 ± 0.8% after 1 h, which dropped to 89.8 ± 2.2% over the subsequent 3 h. The RCP of [^68^Ga]Ga-DOTA-GA-RAD202.1 remained above 99% for all three conditions after 1 h of incubation at 37°C. Even after 4 h, no major changes were observed. The RCP in HS remained 99.5 ± 0.2% for the dilution ratio 1/1 and 98.6 ± 0.6% for the dilution ratio 1/9. The lowest RCP was measured in PBS 1/9 after 4 h with 97.7 ± 0.2% (Figure S3H).

***In vitro* cell binding affinity of** **[^68^Ga]Ga-DOTA-GA-RAD202 and [^68^Ga]Ga-DOTA-GA-RAD202.1**

The uptake of [^68^Ga]Ga-DOTA-GA-RAD202 and [^68^Ga]Ga-DOTA-GA-RAD202.1 was investigated in three HER2-positive cell lines, SK-BR-3, SKOV-3 and BT474, and one HER2-negative cell line MDA-MB231 after 1 h and 4 h of incubation.

For [^68^Ga]Ga-DOTA-GA-RAD202 (Figure S3I), HER2-positive cells showed significantly higher uptake than MDA‑MB231 cells at both time points. SK-BR-3 cells exhibited the highest uptake (59 ± 7.6% applied dose (AD)/mg at 1 h, increasing to 74.2 ± 9.9% AD/mg at 4 h). The significantly lowest cell uptake among the HER2‑positive cell lines was measured in SKOV-3 cells for both time points. Here, the uptake after 1 h was 30.1 ± 5.9% AD/mg, and it significantly increased after 4 h up to 35.9 ± 10.8% AD/mg. A time dependent increase in uptake was also observed for BT474 (45.7 ± 10.0% AD/mg vs. 48.7 ± 5.7% AD/mg, after 1 h and 4 h, respectively) and MDA-MB231 cells (2.8 ± 1.6% AD/mg vs. 5.7 ± 1.4% AD/mg, after 1 h and 4 h, respectively). Blocking tests reduced uptake in HER2-positive cells, however this effect was not significant in MDA-MB231 cells.

For [^68^Ga]Ga-DOTA-GA-RAD202.1 (Figure S3J), uptake in HER2-positive cells was significantly higher than in MDA-MB231 cells, with the highest levels detected in SK-BR-3 cells (72.8 ± 9.4% AD/mg at 1 h, 41.5 ± 6.2% AD/mg at 4 h). The HER2-positive cells also exhibited a significant decrease in cell uptake at both time points following the addition of unlabeled sdAb. None of the four cell lines showed a significant time dependent increase of tracer binding.

[^68^Ga]Ga-DOTA-GA-RAD202.1 exhibited a higher affinity for the HER2 receptor after 1 h and 4 h of incubation compared to the HIS-tag conjugate [^68^Ga]Ga-DOTA-GA-RAD202. This correlates with findings from previous studies, which showed that the position of the HIS-tag could affect the binding properties. Especially His-tags placed at the C-terminus, which is also the case for NM-02, can adversely impact binding affinity, while N-terminal HIS-tag may better maintain binding characteristics [2]. In addition, the cell uptake rates, especially of [^68^Ga]Ga-DOTA-GA-RAD202, correspond to the HER2 levels in the applied cell lines [3].


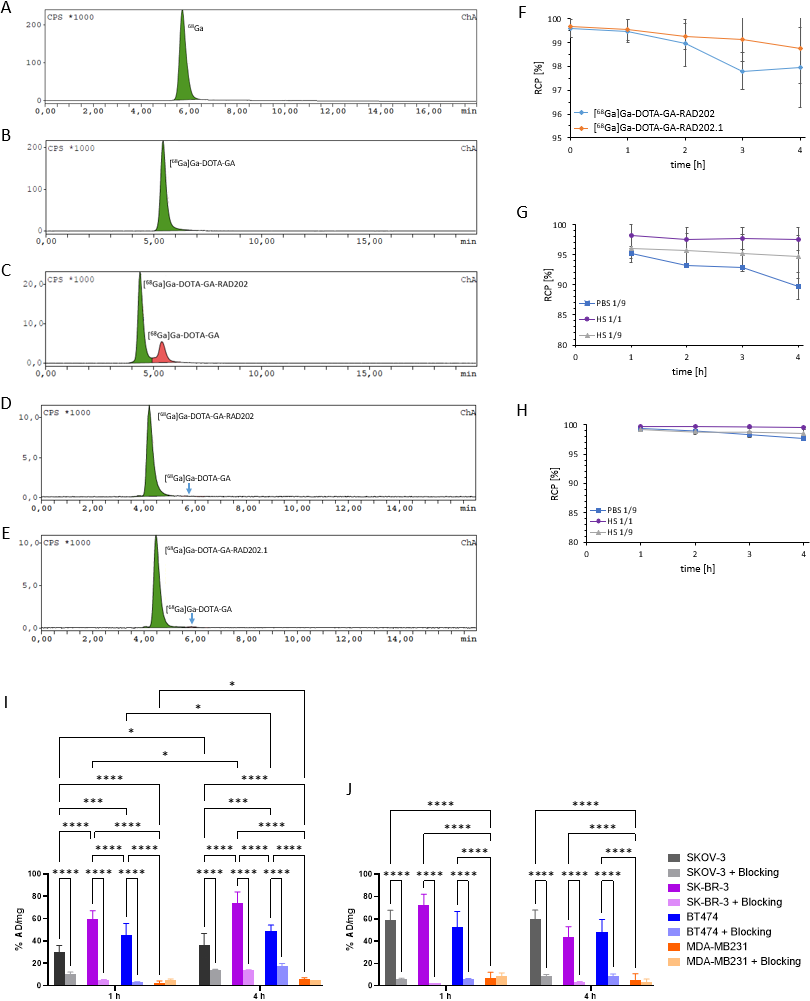


Figure S3: Quality control, stability and cell uptake of [^68^Ga]Ga-DOTA-GA-RAD202 and [^68^Ga]Ga-DOTA-GA-RAD202.1

Representative HPLC chromatograms for (A) ^68^Ga, (B) ^68^Ga-DOTA-GA, (C) [^68^Ga]Ga-DOTA-GA-RAD202 before purification and purified **(D)** [^68^Ga]Ga-DOTA-GA-RAD202 and **(E)** [^68^Ga]Ga-DOTA-GA-RAD202.1. **(F)** Stability of [^68^Ga]Ga-DOTA-GA-RAD202 (blue) and [^68^Ga]Ga-DOTA-GA-RAD202.1 (orange) in saline at room temperature (25°C) up to 4 h post labeling and (G-H) in phosphate-buffered saline (PBS) in 1/9 dilution ratio (blue line) in and human serum (HS) in 1/1 (purple line) and 1/9 dilution ratios (grey line) at 37°C up to 4 h for **(G)** [^68^Ga]Ga-DOTA-GA-RAD202 and **(H)** [^68^Ga]Ga-DOTA-GA-RAD202.1. Radiochemical purities (RCP) as a function of time. Data are presented as mean ± SD (n = 3). (I-J) The cell uptake of **(I)** [^68^Ga]Ga-DOTA-GA-RAD202 and **(J)** [^68^Ga]Ga-DOTA-GA-RAD202.1 was investigated in HER2-positive (SKOV-3, SK‑BR‑3 and BT474) and a HER2-negative cell line (MDA‑MB231) after 1 h and 4 h and is depicted as percent uptake of applied dose (AD) per mg protein. A 100-fold molar excess of non-labeled single domain antibody was used for blocking of the HER2 receptors. Data are presented as mean ± SD (n ≤ 11). Significance was tested using two-way ANOVA with Tukey’s post-hoc test (**** p ≤ 0.0001; *** p ≤ 0.001; * p ≤ 0.05; n.s. p > 0.05). AD: applied dose; HS: human serum; PBS: phosphate-buffered saline; RCP: radiochemical purity.


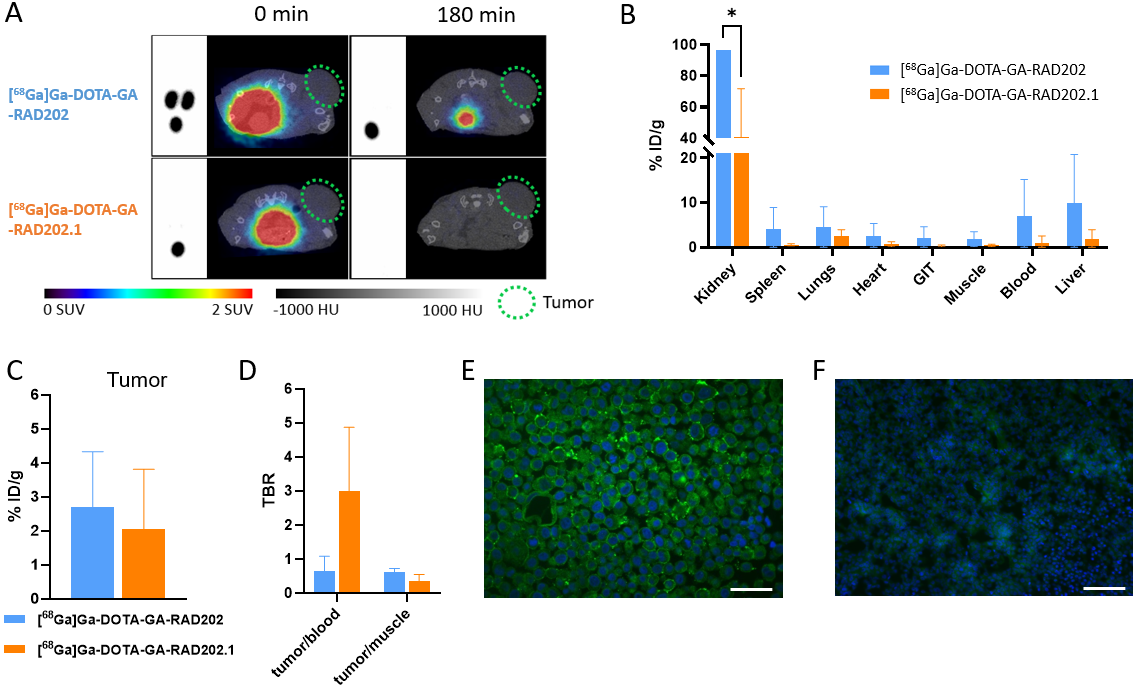


**Figure S4:** **PET/CT imaging of [^68^Ga]Ga-DOTA-GA-RAD202 and [^68^Ga]Ga-DOTA-GA-RAD202.1 in a SK‑BR-3 xenograft mouse model.**

Representative maximum intensity projections and transaxial PET/CT images of SK-BR-3 xenograft mice injected with [^68^Ga]Ga-DOTA-GA-RAD202 and [^68^Ga]Ga-DOTA-GA-RAD202.1 immediately after injection and 3 h post injection (p.i.). The images were acquired after intravenous injection of 10 ± 1 MBq [^68^Ga]Ga-DOTA-GA-RAD202 and [^68^Ga]Ga-DOTA-GA-RAD202.1 and Gelofusine® as kidney protector. The CT images are scaled between -1000 Hounsfield unit (HU) and 1000 HU, while PET images are scaled up to two standard uptake value (SUV). **(B)** *Ex vivo* analysis after the last PET/CT (4 h p.i.) of the harvested organs and **(C)** the tumor are represented as % injected dose (ID)/g tissue. **(D)** The tumor to background (TBR) with blood or muscle taken as background. Representative fluorescence microscopy image of HER2 staining of SK-BR-3 cells **(E)** *in vitro* and **(F)** SK-BR-3 xenograft sections *ex vivo*. Staining with anti-HER2 (green) and DAPI (blue) for the nuclei. 20x magnification; scale bar: 100 μm. All data are presented as mean ± SD (n ≤ 3). Significance was tested using two-way ANOVA and Tukey’s post-hoc test (B) (* p ≤ 0.05; n.s. p > 0.05). GIT = gastro intestinal tract.


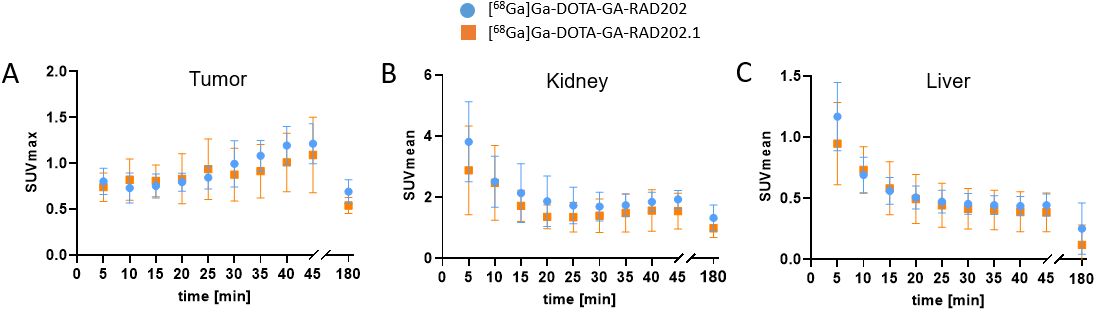


**Figure S5: PET/CT imaging of [^68^Ga]Ga-DOTA-GA-RAD202 and [^68^Ga]Ga-DOTA-GA-RAD202.1 in HER2-positive SKOV-3 xenograft model.**

The maximal SUV for **(A)** the tumor, the mean SUVs for **(B)** the kidney and (C) liver for HER2-positive SKOV‑3 xenograft mice injected with [^68^Ga]Ga-DOTA-GA-RAD202 and [^68^Ga]Ga-DOTA-GA-RAD202.1. Data are presented as mean ± SD (n = 6).

**Table S1: SUV values from PET/CT imaging with [^68^Ga]Ga-DOTA-GA-RAD202 and [^68^Ga]Ga-DOTA-GA-RAD202.1 in HER2-positive SKOV-3 xenograft model**

The maximal and mean SUV for the tumor, the mean SUVs for the kidney and liver for HER2-positive SKOV‑3 xenograft mice injected with [^68^Ga]Ga-DOTA-GA-RAD202 and [^68^Ga]Ga-DOTA-GA-RAD202.1. Data are presented as mean ± SD (n = 6)

|  | **SUVmax Tumor** | | **SUVmean Tumor** | | **SUVmean Kidney** | | **SUVmean Liver** | |
| --- | --- | --- | --- | --- | --- | --- | --- | --- |
| Time [min] | [^68^Ga]Ga-DOTA-GA-RAD202 | [^68^Ga]Ga-DOTA-GA-RAD202.1 | [^68^Ga]Ga-DOTA-GA-RAD202 | [^68^Ga]Ga-DOTA-GA-RAD202.1 | [^68^Ga]Ga-DOTA-GA-RAD202 | [^68^Ga]Ga-DOTA-GA-RAD202.1 | [^68^Ga]Ga-DOTA-GA-RAD202 | [^68^Ga]Ga-DOTA-GA-RAD202.1 |
| 5 | 0.8 ± 0.1 | 0.7 ± 0.1 | 0.6 ± 0.2 | 0.4 ± 0.0 | 3.8 ± 1.1 | 2.9 ± 1.3 | 1.2 ± 0.3 | 0.9 ± 0.3 |
| 10 | 0.7 ± 0.1 | 0.8 ± 0.2 | 0.5 ± 0.2 | 0.4 ± 0.0 | 2.5 ± 0.7 | 2.5 ± 1.1 | 0.7 ± 0.1 | 0.7 ± 0.2 |
| 15 | 0.8 ± 0.1 | 0.8 ± 0.1 | 0.5 ± 0.2 | 0.4 ± 0.1 | 2.1 ± 0.8 | 1.7 ± 0.5 | 0.6 ± 0.1 | 0.6 ± 0.2 |
| 20 | 0.8 ± 0.1 | 0.8 ± 0.2 | 0.5 ± 0.2 | 0.4 ± 0.1 | 1.9 ± 0.7 | 1.4 ± 0.3 | 0.5 ± 0.1 | 0.5 ± 0.2 |
| 25 | 0.8 ± 0.1 | 0.9 ± 0.3 | 0.5 ± 0.2 | 0.4 ± 0.1 | 1.7 ± 0.5 | 1.3 ± 0.4 | 0.5 ± 0.1 | 0.4 ± 0.2 |
| 30 | 1.0 ± 0.2 | 0.9 ± 0.2 | 0.6 ± 0.2 | 0.4 ± 0.1 | 1.7 ± 0.4 | 1.4 ± 0.5 | 0.5 ± 0.1 | 0.4 ± 0.1 |
| 35 | 1.1 ± 0.1 | 0.9 ± 0.2 | 0.6 ± 0.2 | 0.4 ± 0.1 | 1.7 ± 0.3 | 1.5 ± 0.6 | 0.4 ± 0.1 | 0.4 ± 0.1 |
| 40 | 1.2 ± 0.2 | 1.0 ± 0.3 | 0.6 ± 0.2 | 0.4 ± 0.1 | 1.8 ± 0.3 | 1.6 ± 0.6 | 0.4 ± 0.1 | 0.4 ± 0.1 |
| 45 | 1.2 ± 0.2 | 1.1 ± 0.3 | 0.6 ± 0.2 | 0.5 ± 0.1 | 1.9 ± 0.3 | 1.5 ± 0.5 | 0.4 ± 0.1 | 0.4 ± 0.1 |
| 180 | 0.7 ± 0.1 | 0.5 ± 0.1 | 0.5 ± 0.1 | 0.5 ± 0.2 | 1.3 ± 0.4 | 1.0 ± 0.3 | 0.3 ± 0.2 | 0.1 ± 0.2 |

PET analysis showed reduced tumor uptake of both radiotracers when co-injected with excess unlabeled sdAb-chelator conjugates (Figure S6A). After 180 min the accumulation in the tumor was reduced by 65.1% in the blocking study with [^68^Ga]Ga-DOTA-GA-RAD202 (Figure S7A). The subsequent *ex vivo* gamma counter analysis confirmed that the co-injection of unlabeled sdAb-chelator conjugates led to a significant decrease in tracer accumulation in the tumor by 55.5% (Figure S6B). For [^68^Ga]Ga-DOTA-GA-RAD202.1, tumor uptake decreased by 33% in PET analysis and 59.2% in *ex vivo* gamma counter analysis (Figure S6B-C). The initial renal uptake of both tracers was lower in the blocking study than in their respective biodistribution studies (2.4±1.1 SUV_mean_ vs. 3.8±1.1 SUV_mean_ and 1.7±0.8 SUV_mean_ vs. 2.9±1.3 SUV_mean_, for [^68^Ga]Ga-DOTA-GA-RAD202 and [^68^Ga]Ga-DOTA-GA-RAD202.1, respectively) (Figure S7B&E). However, the uptake decreased only slowly, so that after 180 min a similar renal uptake was observed as in the biodistribution study (1.3±0.4 SUV_mean_ vs. 1.3±0.4 SUV_mean_ and 0.8±0.1 SUV_mean_ vs. 1.0±0.3 SUV_mean_, for [^68^Ga]Ga-DOTA-GA-RAD202 and [^68^Ga]Ga-DOTA-GA-RAD202.1, respectively). *Ex vivo* analysis confirmed significantly reduced kidney uptake in the blocking studies for [^68^Ga]Ga-DOTA-GA-RAD202 (74.8±2.6% vs. 91.9±1.6 ID/g) and [^68^Ga]Ga-DOTA-GA-RAD202.1 (23.6±16.1% vs. 44.7±11.0% ID/g). Liver uptake showed a slow decrease in blocking studies. [^68^Ga]Ga-DOTA-GA-RAD202 uptake declined from 0.9±0.2 SUV_mean_ to 0.5±0.4 SUV_mean_ over 180 min, while PET imaging at 30 min p.i. and *ex vivo* analyses indicated higher uptake compared to the biodistribution study (Figure S7C&F). By co-injection of unlabeled ‘cold’ sdAb-chelator conjugates with [^68^Ga]Ga-DOTA-GA-RAD202.1, the uptake in the liver dropped from 0.6±0.3 SUV_mean_ to 0.2±0.3 SUV_mean_, matching biodistribution results. Consistent with these results, e*x vivo* findings confirmed similar liver uptake between biodistribution and blocking studies for [^68^Ga]Ga-DOTA-GA-RAD202.1 (2.3±1.2%ID/g vs. 2.4±0.1% ID/g; Figure S6C).


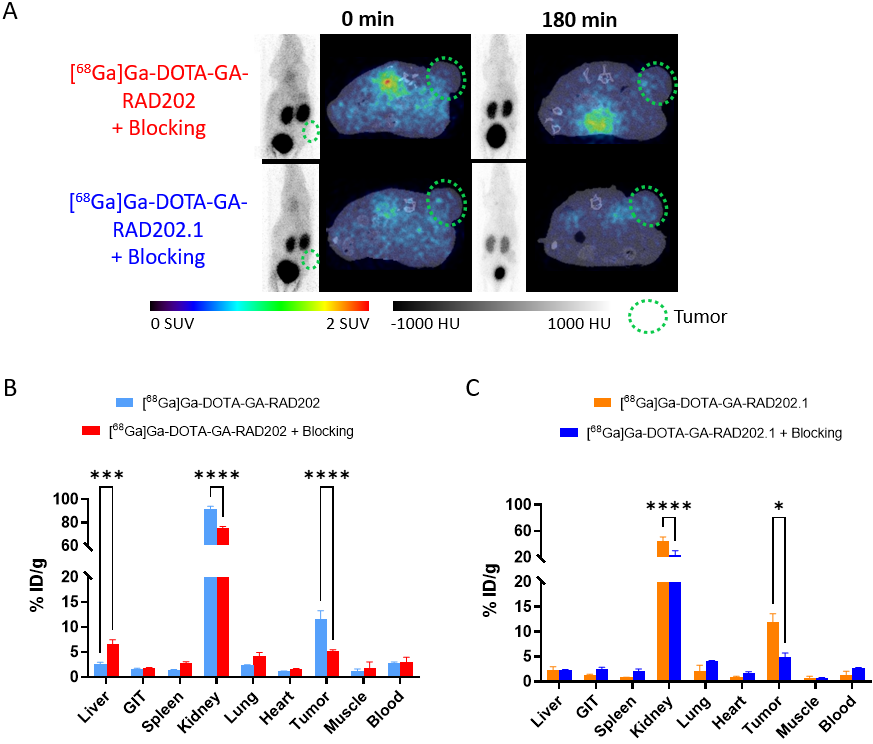


Figure S6: PET/CT imaging of blocking study with [^68^Ga]Ga-DOTA-GA-RAD202 and [^68^Ga]Ga-DOTA-GA-RAD202.1 in HER2-positive SKOV-3 xenograft model.

(A) Representative maximum intensity projections and transaxial PET/CT images of HER2-positive SKOV-3 xenograft mice injected with [^68^Ga]Ga-DOTA-GA-RAD202 or [^68^Ga]Ga-DOTA-GA-RAD202.1 and co-injection of unlabeled sdAb-chelator conjugates 0 min and 180 min post injection. The CT images are scaled between -1000 HU and 1000 HU, while PET images are scaled up to two standard uptake value (SUV). (B-C) *Ex vivo* analysis after the last PET/CT (4 h p.i.) of the harvested organs are represented as % injected dose (ID)/g tissue. Data are presented as mean ± SD (n=3). Significance was tested using two-way ANOVA and Tukey’s post-hoc test (**** p ≤ 0.0001; *** p ≤ 0.001; * p ≤ 0.05; n.s. p > 0.05). GIT: gastro intestinal tract.


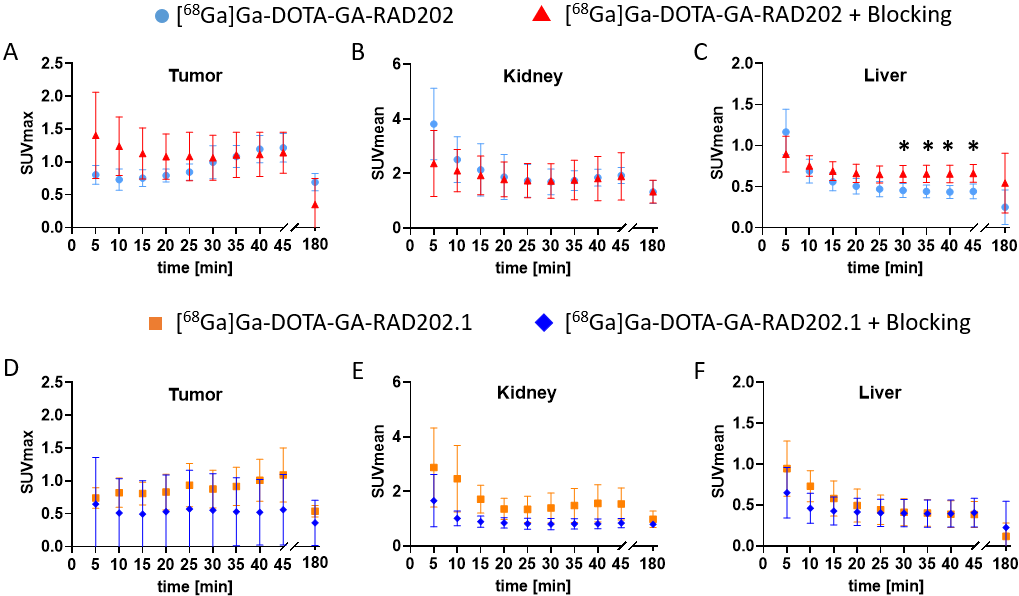


**Figure S7: PET/CT imaging of blocking study with [^68^Ga]Ga-DOTA-GA-RAD202 and [^68^Ga]Ga-DOTA-GA-RAD202.1 in HER2-positive SKOV-3 xenograft model.**

The maximal SUV for **(A & D)** the tumor, the mean SUVs for **(B & E)** the kidney and **(C & F)** liver are presented as a function of time for HER2-positive SKOV-3 xenograft mice injected with [^68^Ga]Ga-DOTA-GA-RAD202 or [^68^Ga]Ga-DOTA-GA-RAD202.1 and co-injection of unlabeled sdAb-chelator conjugates. Data are presented as mean ± SD (n = 6). Significance was tested using two-way ANOVA and Tukey’s post-hoc test (* p ≤ 0.05; n.s. p > 0.05).

**Synthesis, purity and stability studies with [^177^Lu]Lu-DOTA-GA-RAD202.1**

The sdAb-chelators were radiolabeled with ^177^Lu with an overall RCY of 85.5 ± 2.9% for the sdAb-chelators without HIS-tag ([^177^Lu]Lu-DOTA-GA-RAD202.1). An exemplary TLC chromatogram of purified product is shown in Figure S7A, demonstrating a radiochemical puritiy of > 99%. The radiolabeled sdAb have R_f_ values between 0 and 0.2, while the small sized free radionuclide ions migrate as citrate complexes with the solvent front with R_f_ values between 0.9 and 1.

To assess the stability of [^177^Lu]Lu-DOTA-GA-RAD202.1 the product was stored in saline at room temperature (25 °C) or in PBS or HS at 37 °C and samples were analyzed with TLC. Figure S7B illustrates that a RCP above 99% was maintained in saline at 25°C for up to 144 h. The product was also able to maintain a purity of 99% in HS over a period of 144 h after end of synthesis. Only when the product was diluted with PBS the stability decreased after 48 h and reached a purity of 90.9 ± 1% after 144 h (Figure S7C).

***In vitro* cell binding affinity of [^177^Lu]Lu-DOTA-GA-RAD202.1**

Figure S7D demonstrates that the uptake of [^177^Lu]Lu-DOTA-GA-RAD202.1 in all HER2-positive cell lines was significantly higher than in the HER2-negative cell line at both time points. The significantly highest uptake was measured in the SK-BR-3 cell line with 77.9 ± 4.4% AD/mg after 1 h and 44.6 ± 10.6% AD/mg after 4 h of incubation. The significantly lowest uptake of [^177^Lu]Lu-DOTA-GA-RAD202.1 among the HER2-expressing cell lines was observed in the SKOV-3 cell line at both time points (18.1 ± 0.9% AD/mg and 17.9 ± 5.4% AD/mg). None of the cell lines showed a significant change in cell uptake over the time except for SK-BR-3 which showed a decrease in tracer uptake over time.

The blocking test with additionally added non-radiolabeled chelator-free NM-02.1, showed a significant decrease in tracer uptake in HER2-positive cells at both time points. No significant difference was detected for the HER2-negative cell line for the two time points.

Although, the cell line SK-BR-3 expresses the highest HER2 level *in vitro*, followed by BT474 and SKOV-3, the SK-BR-3 cell line showed a decrease in uptake between 1 h and 4 h incubation with the tracers [^68^Ga]Ga-DOTA-GA-RAD202, [^177^Lu]Lu-DOTA-GA-RAD202 (not shown) and [^177^Lu]Lu-DOTA-GA-RAD202.1. This could be due to the rapid antibody-induced internalization and efficient degradation of HER2 receptors in this cell line. Normally, constitutive internalization of HER2 and membrane recycling occur at a very slow rate in many cell systems in the absence of a recognized agonist. However, several research groups presented HER2 internalization induced by a single mAb or a combination of multiple HER2 specific mAbs even after an incubation for 4 h.

**Cytotoxic effect of [^177^Lu]Lu-DOTA-GA-RAD202.1**

The XTT cell viability assay was performed to assess cytotoxic effects of increasing amounts of [^177^Lu]Lu-DOTA-GA-RAD202.1 on human cancer cell lines. To compare the results, the half-maximal inhibitory concentration (IC_50_) was determined, which indicates the concentration (here: radioactivity) of a drug required to inhibit a biochemical process, in this case the cell proliferation, by 50%. Figure S7E shows the cell viability as a function of the administered amount of [^177^Lu]Lu-DOTA-GA-RAD202.1. The SK-BR-3 cell line was significantly more sensitive than the other HER2-positive cell lines with an IC_50_ of 0.30 ± 0.08 MBq. The BT474 cell line, on the other hand, was the significantly most resistant cell line among all investigated cell lines with a calculated IC_50_ of 0.66 ± 0.05 MBq. Interestingly, the HER2-negative cell line MDA‑MB231 showed a similar sensitivity to [^177^Lu]Lu-DOTA-GA-RAD202.1 as the SK-BR-3 cell line with an IC_50_ value of 0.29 ± 0.11 MBq (Figure S7F).


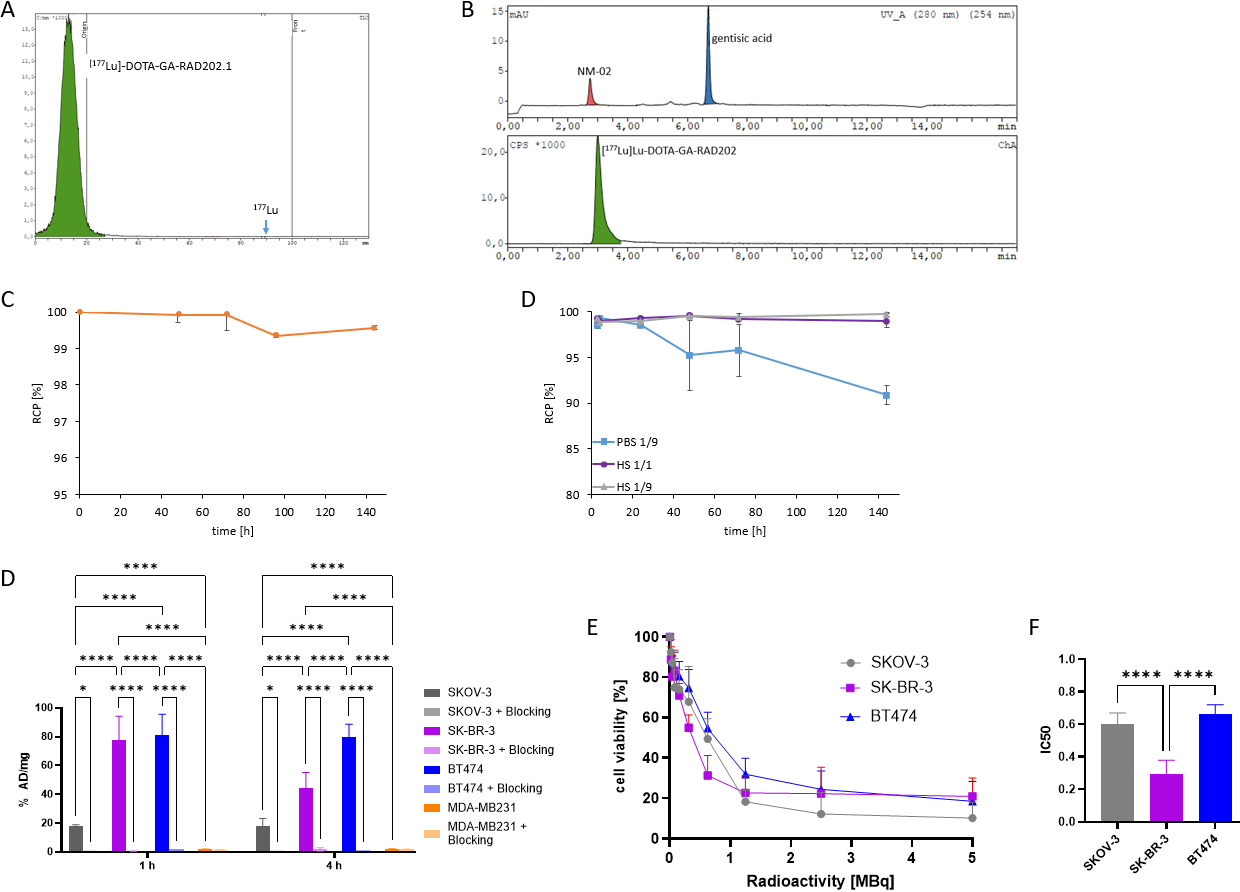


**Figure S8: Quality control, stability, cell uptake and cytotoxicity of [^177^Lu]Lu-DOTA-GA-RAD202.1**

**(A)** Representative TLC chromatograms for purified [^177^Lu]Lu-DOTA-GA-RAD202.1. Radiation detector counts are plotted against the ascending solvent (citrate) distance on the TLC plate. The R_f_ for the product was 0.16, and the radiochemical purity 99.9%. **(B)** Stability of [^177^Lu]Lu-DOTA-GA-RAD202.1 in saline at room temperature (25°C) up to 144 h post labeling and **(C)** in phosphate-buffered saline (PBS) in 1/9 dilution ratio (blue line) and in human serum (HS) in 1/1 (purple line) and 1/9 dilution ratios (grey line) at 37°C up to 144 h. Radiochemical purity (RCP) as a function of time. Data are presented as mean ± SD (n = 3). The cell uptake of **(D)** [^177^Lu]Lu-DOTA-GA-RAD202.1 was investigated in HER2-positive (SKOV-3, SK‑BR‑3 and BT474) and HER2-negative cell line (MDA‑MB231) after 1 h and 4 h and is depicted as percent uptake of applied dose (AD) per mg protein. A 100-fold molar excess of non-labeled single domain antibody was used for blocking of the HER2 receptors. Data are presented as mean ± SD (n ≤ 11). Significance was tested using two-way ANOVA with Tukey’s post-hoc test. **(E)** Cell lines that showed tracer uptake (SKOV-3, SK‑BR‑3 and BT474) were treated with ten different amounts of [^177^Lu]Lu-DOTA-GA-RAD202.1 for 72 h and cell viability was then evaluated using the XTT assay. Cell viability in the absence of treatment was set at 100%. **(F)** The inhibitory concentration (IC_50_) values of investigated cell lines. Data are presented as mean ± SD (n = 6). Significance was tested using one-way ANOVA with Tukey’s post-hoc test (**** p ≤ 0.0001; *** p ≤ 0.001; * p ≤ 0.05; n.s. p > 0.05). AD: applied dose; HS: human serum; IC_50_: inhibitory concentration; PBS: phosphate-buffered saline; RCP: radiochemical purity.


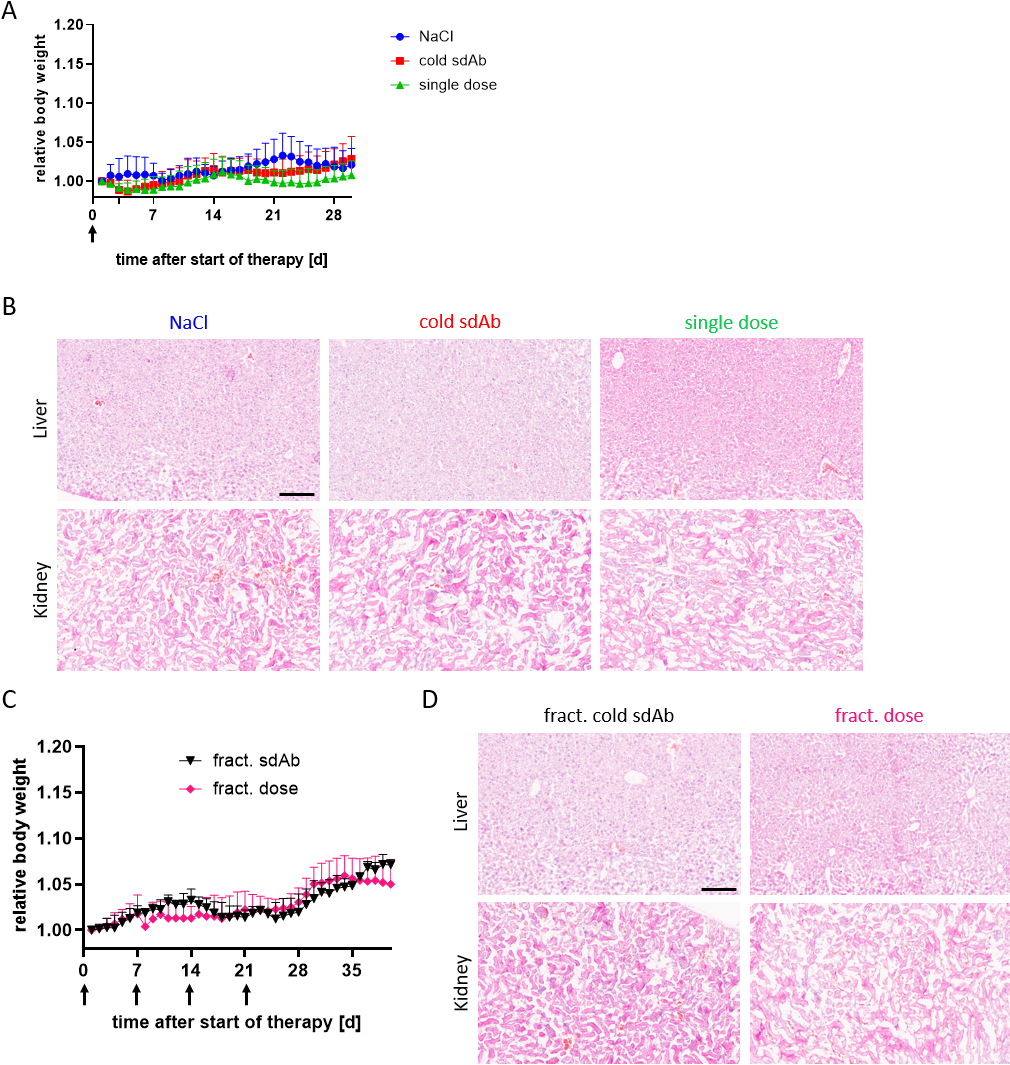


**Figure S9:** **Change of bodyweight and effect on non-target organs with single dose or fractionated therapy**

**(A)** The relative bodyweight of the mice after starting the treatment. The arrow indicates the time point of injection of either NaCl (blue), cold sdAb-chelator conjugate (red) or [^177^Lu]Lu-DOTA-GA-RAD202.1 as single dose (green). **(B)** Haematoxylin-Eosin staining of the liver and kidney for the three treatment groups in single cycle therapy. **(C)** The relative bodyweight of the mice after starting the fractionated therapy. The arrows indicate the timepoints of injection of either unlabeled ‘cold’ sdAb-chelator conjugate (black) or [^177^Lu]Lu-DOTA-GA-RAD202.1 (pink). **(D)** Haematoxylin-Eosin staining of the liver and kidney for both treatment groups. 10x magnification; scale bar: 100 μm


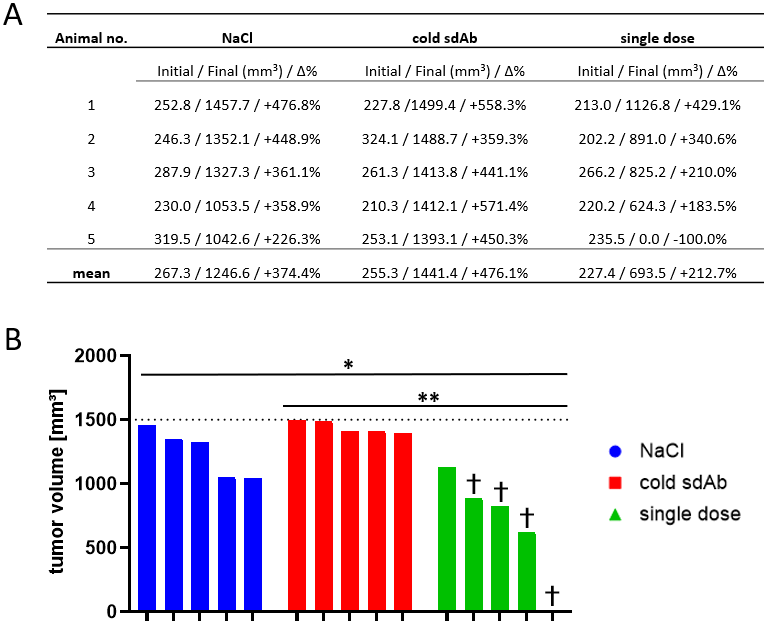


**Figure S10: Effect of single cycle therapy with [^177^Lu]Lu-DOTA-GA-RAD202.1**

(A) For each animal, the initial and final tumor volumes (in mm³) are listed, along with the corresponding percentage change (∆%). A Δ of –100% indicates that the tumor has completely disappeared. (B) Waterfall plot analysis of tumor volumes on the day of killing for every single mouse. Data are presented as mean ± SD (n = 5). Significances were test-ed with Welch ANOVA with Brown-Forsythe post-hoc test. (** p ≤ 0.01; * p ≤ 0.05; n.s. p > 0.05). ✝ = The cause of killing was the end of observation period.


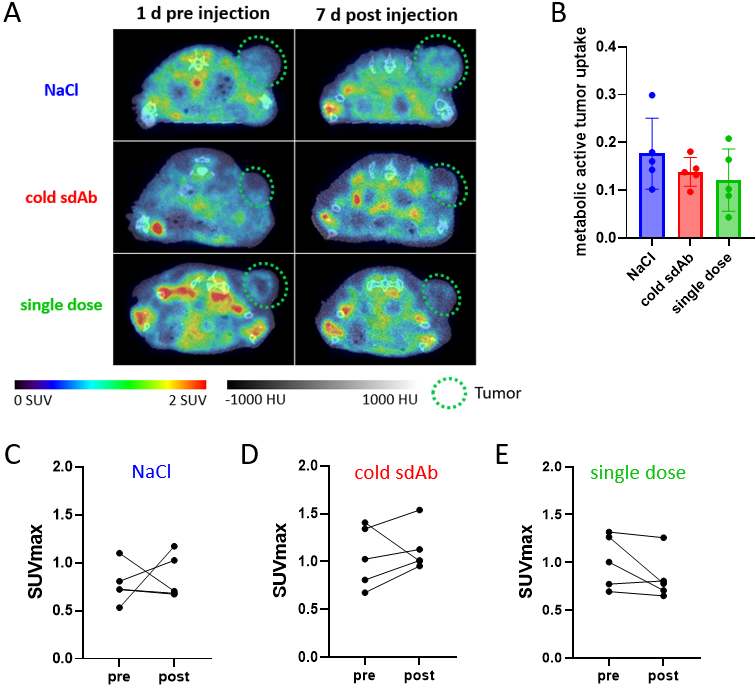


**Figure S11:** **[^18^F]FDG uptake one day before and seven days after the single cycle therapy**

Representative transaxial PET/CT images of [^18^F]FDG distribution in HER2-positive SKOV-3 xenograft mice injected with 0.9% NaCl, unlabeled ‘cold’ sdAb-chelator conjugate or [^177^Lu]Lu-DOTA-GA-RAD202.1 one day before (pre) and seven days after (post) the start of therapy. The CT images are scaled between -1000 Hounsfield unit (HU) and 1000 HU, while PET images are scaled up to two standard uptake value (SUV). **(B)** Standardized metabolic active tumor uptake seven days after the injection and paired comparison of SUV_max_ from pre to post injection for the mice injected with **(C)** NaCl, **(D)** cold sdAb-chelator conjugate and **(E)** single dose of [^177^Lu]Lu-DOTA-GA-RAD202.1. Significances were test-ed with Welch ANOVA with Brown-Forsythe post-hoc test (B) or non-parametric paired t-test (C-E). (n.s. p > 0.05). FDG: fluorodeoxyglucose; HU: hounsfield unit; SUV: standard uptake value.


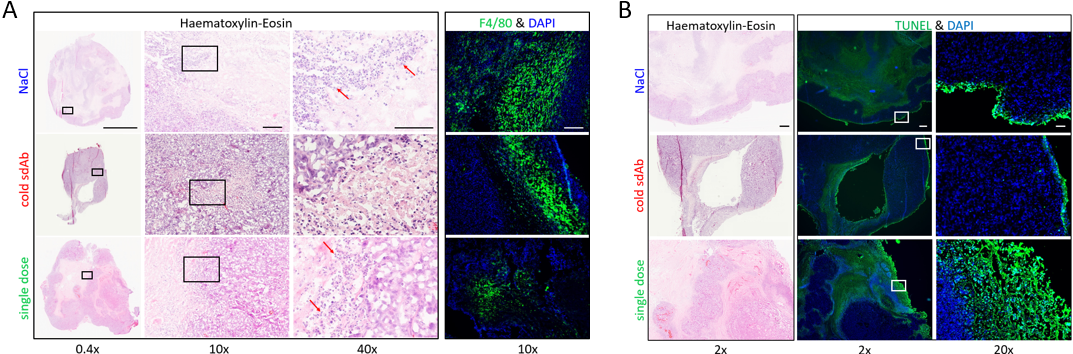


Figure S12: Microscopic evaluation of tumor tissue from mice in single cycle therapy

**(A)** Different magnifications (0.4x, 10x & 40x) of HE staining of the tumor of mice injected with 0.9% NaCl, unlabeled ‘cold’ sdAb-chelator conjugate or single dose of [^177^Lu]Lu-DOTA-GA-RAD202.1. The red arrows exemplary point to leukocytes. Representative fluorescence microscopy images for F4/80 staining (green) for identification of macrophages. Scale bar 0.4x: 5 mm; 10x: 200 μm; 40x: 100 μm. **(B)** HE stainings and corresponding fluorescence microscopy images for TUNEL staining (green) for identification of apoptotic cells. Scale bar 2x: 500 μm; 20x: 200 μm. Nuclei were counterstained with DAPI (blue). The black/white box inside the tumor tissue demonstrates the section in the next higher magnification.


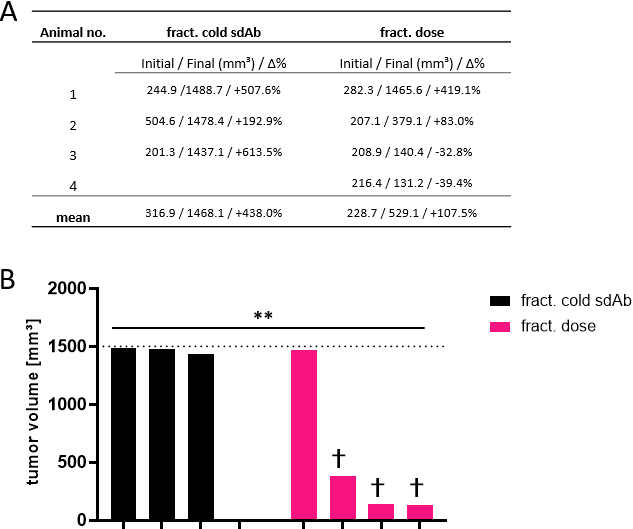


**Figure S13: Effect of fractionated therapy with [^177^Lu]Lu-DOTA-GA-RAD202.1**

(A) For each animal, the initial and final tumor volumes (in mm³) are listed, along with the corresponding percentage change (∆%). A negative Δ indicates tumor regression. (B) Waterfall plot analysis of tumor volumes on the day of killing for every single mouse in fractionated therapy. Significances weas tested using unpaired t-test with Welch’s correction (** p ≤ 0.01; n.s. p > 0.05). ✝ = The cause of finalization was the end of observation period.


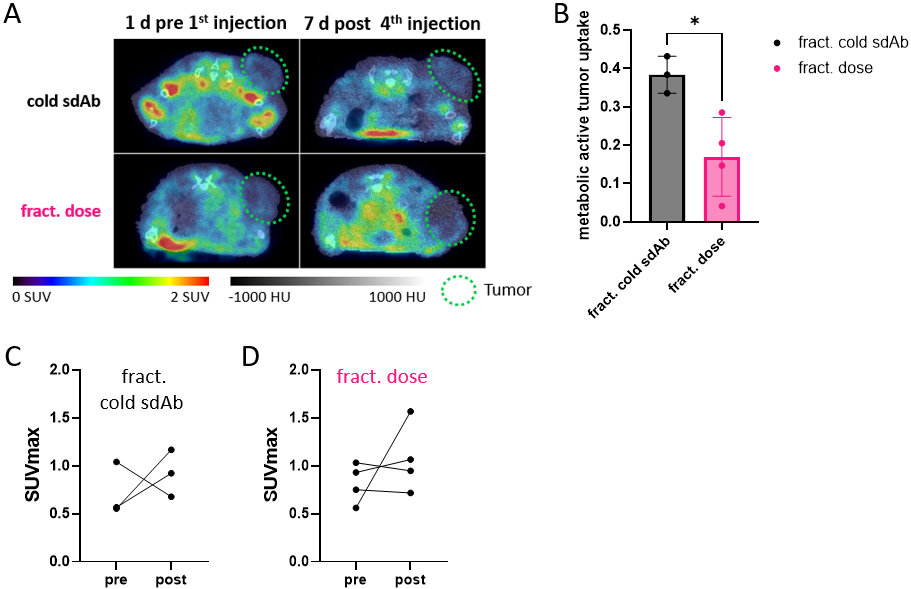


**Figure S14:** **[^18^F]FDG uptake one day before the first and seven days after the fourth injection**

Representative transaxial PET/CT images of [^18^F]FDG distribution in HER2-positive SKOV-3 xenograft mice injected with four doses of unlabeled ‘cold’ sdAb-chelator conjugate or [^177^Lu]Lu-DOTA-GA-RAD202.1 one day before the first injection (pre) and seven days after the fourth injection (post). The CT images are scaled between -1000 Hounsfield unit (HU) and 1000 HU, while PET images are scaled up to two standard uptake value (SUV). **(B)** Standardized metabolic active tumor uptake seven days after the fourth injection and paired comparison of SUV_max_ from pre to post injection for mice injected with **(C)** cold sdAb-chelator conjugate and **(D)** fractionated dose of [^177^Lu]Lu-DOTA-GA-RAD202.1. Significances were tested using unpaired t-test with Welch’s correction (B) or nonparametric paired t-test (C, D) (* p ≤ 0.05; n.s. p > 0.05). FDG: fluorodeoxyglucose; HU: hounsfield unit; SUV: standard uptake value.


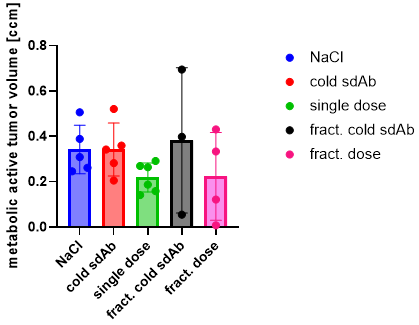


**Figure S15: Standardized metabolic active tumor volume obtained through [^18^F]FDG measurements**

Standardized metabolic active tumor volume seven days after the injection of a single dose with NaCl (blue), cold sdAb-chelator conjugate (red), [^177^Lu]Lu-DOTA-GA-RAD202.1 (green) and seven days after the fourth injection with cold sdAb-chelator conjugate (grey) or [^177^Lu]Lu-DOTA-GA-RAD202.1 (pink). Data are presented as mean ± SD (n ≤ 5). Significances were tested using Welch ANOVA with Brown-Forsythe post-hoc test (n.s. p > 0.05).


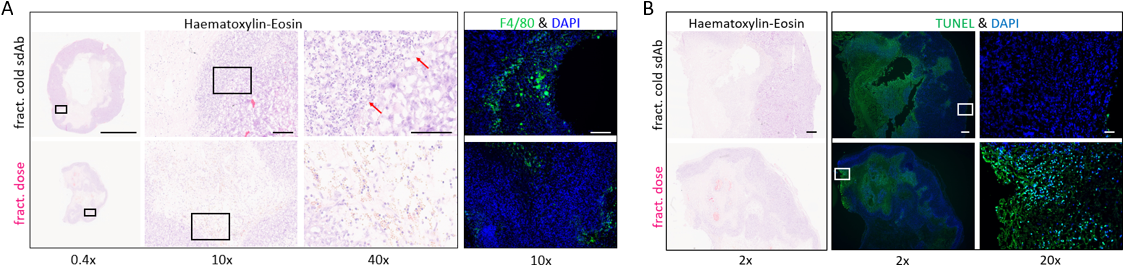


**Figure S16: Microscopic evaluation of tumor tissue from mice in fractionated therapy**

**(A)** Different magnifications (0.4x, 10x & 40x) of HE staining of the tumor of mice injected with four doses of unlabeled ‘cold’ sdAb-chelator conjugate or [^177^Lu]Lu-DOTA-GA-RAD202.1. The red arrows exemplarily point to leukocytes. Representative fluorescence microscopy images for F4/80 staining (green) for identification of macrophages. Scale bar 0.4x: 5 mm; 10x: 200 μm; 40x: 100 μm. **(B)** HE stainings and corresponding fluorescence microscopy images for TUNEL staining (green) for identification of apoptotic cells. Scale bar 2x: 500 μm; 20x: 200 μm. Nuclei were counterstained with DAPI (blue). The black/white box inside the tumor tissue marks the section displayed in the next higher magnification.

**References**

1. D'Huyvetter, M., et al., *Development of 177Lu-nanobodies for radioimmunotherapy of HER2-positive breast cancer: evaluation of different bifunctional chelators.* Contrast Media Mol Imaging, 2012. **7**(2): p. 254-64.

2. Goel, A., et al., *Relative position of the hexahistidine tag effects binding properties of a tumor-associated single-chain Fv construct.* Biochim Biophys Acta, 2000. **1523**(1): p. 13-20.

3. Gall, V.A., et al., *Trastuzumab Increases HER2 Uptake and Cross-Presentation by Dendritic Cells.* Cancer Res, 2017. **77**(19): p. 5374-5383.
